# Human liver single nucleus and single cell RNA sequencing identify a hepatocellular carcinoma-associated cell-type affecting survival

**DOI:** 10.1186/s13073-022-01055-5

**Published:** 2022-05-17

**Authors:** Marcus Alvarez, Jihane N. Benhammou, Nicholas Darci-Maher, Samuel W. French, Steven B. Han, Janet S. Sinsheimer, Vatche G. Agopian, Joseph R. Pisegna, Päivi Pajukanta

**Affiliations:** 1 Department of Human Genetics, David Geffen School of Medicine at UCLA, Los Angeles, CA USA; 2 Vatche and Tamar Manoukian Division of Digestive Diseases, David Geffen School of Medicine at UCLA, Los Angeles, CA USA; 3 Division of Gastroenterology, Hepatology and Parenteral Nutrition, Department of Medicine, VA Greater Los Angeles Healthcare System, Los Angeles, CA USA; 4 Department of Pathology, David Geffen School of Medicine at UCLA, Los Angeles, CA USA; 5 Department of Medicine, David Geffen School of Medicine at UCLA, Los Angeles, CA USA; 6 Department of Computational Medicine, UCLA, Los Angeles, CA USA; 7 Bioinformatics Interdepartmental Program, UCLA, Los Angeles, CA USA; 8 Dumont-UCLA Transplant and Liver Cancer Centers, Department of Surgery, David Geffen School of Medicine at UCLA, Los Angeles, CA USA; 9 Institute for Precision Health, David Geffen School of Medicine at UCLA, Los Angeles, CA USA

## Abstract

**Background:**

Hepatocellular carcinoma (HCC) is a common primary liver cancer with poor overall survival. We hypothesized that there are HCC-associated cell-types that impact patient survival.

**Methods:**

We combined liver single nucleus (snRNA-seq), single cell (scRNA-seq), and bulk RNA-sequencing (RNA-seq) data to search for cell-type differences in HCC. To first identify cell-types in HCC, adjacent non-tumor tissue, and normal liver, we integrated single-cell level data from a healthy liver cohort (*n* = 9 non-HCC samples) collected in the Strasbourg University Hospital; an HCC cohort (*n* = 1 non-HCC, *n* = 14 HCC-tumor, and *n* = 14 adjacent non-tumor samples) collected in the Singapore General Hospital and National University; and another HCC cohort (*n* = 3 HCC-tumor and *n* = 3 adjacent non-tumor samples) collected in the Dumont-UCLA Liver Cancer Center. We then leveraged these single cell level data to decompose the cell-types in liver bulk RNA-seq data from HCC patients’ tumor (*n* = 361) and adjacent non-tumor tissue (*n* = 49) from the Cancer Genome Atlas (TCGA) multi-center cohort. For replication, we decomposed 221 HCC and 209 adjacent non-tumor liver microarray samples from the Liver Cancer Institute (LCI) cohort collected by the Liver Cancer Institute and Zhongshan Hospital of Fudan University.

**Results:**

We discovered a tumor-associated proliferative cell-type, Prol (80.4% tumor cells), enriched for cell cycle and mitosis genes. In the liver bulk tissue from the TCGA cohort, the proportion of the Prol cell-type is significantly increased in HCC and associates with a worse overall survival. Independently from our decomposition analysis, we reciprocally show that Prol nuclei/cells significantly over-express both tumor-elevated and survival-decreasing genes obtained from the bulk tissue. Our replication analysis in the LCI cohort confirmed that an increased estimated proportion of the Prol cell-type in HCC is a significant marker for a shorter overall survival. Finally, we show that somatic mutations in the tumor suppressor genes *TP53* and *RB1* are linked to an increase of the Prol cell-type in HCC.

**Conclusions:**

By integrating liver single cell, single nucleus, and bulk expression data from multiple cohorts we identified a proliferating cell-type (Prol) enriched in HCC tumors, associated with a decreased overall survival, and linked to *TP53* and *RB1* somatic mutations.

**Supplementary Information:**

The online version contains supplementary material available at 10.1186/s13073-022-01055-5.

## Background

Hepatocellular carcinoma (HCC) is the third leading cause of cancer-related death world-wide [1]. Although early detection has been associated with improved overall survival [2], most patients present in later stages, which prevents curative therapies such as hepatic resection and liver transplantation, resulting in a 5-year survival of only 18% [3]. Previous studies have demonstrated that tumor heterogeneity is common in HCC [4], which may explain some of the differences in survival outcomes and responses to therapies [5, 6]. Sub-classification of HCCs by molecular and cellular characteristics could help guide biomarker discovery and treatment options, especially in NAFLD-related HCCs, which remain poorly understood and underrepresented in most transcriptomic HCC studies.

Single-cell RNA sequencing (scRNA-seq) has advanced the study of complex admixtures of cells, shedding light on cellular functions at the single cell level in unprecedented ways [7–10]. However, applying scRNA-seq technology to precious, archived human tissues, such as liver biopsies or resections, has proven to be challenging as it is not possible to dissociate intact cells from these existing biopsies of solid tissues. Single nucleus RNA sequencing (snRNA-seq) techniques [11] have overcome these technical challenges [12] and enabled cell-type level characterization of frozen solid tissues [13–16]. As scRNA-seq and snRNA-seq technologies improve, their use for solid tissues, such as liver, has expanded [17, 18]. However, studies integrating data from multiple single cell level cohorts are needed to improve power of small individual cohorts.

In the field of tumor biology, scRNA-seq and snRNA-seq have helped elucidate the presence of tumor heterogeneity, which is commonly observed at the molecular and clinical level in HCC [19–21]. ScRNA-seq and snRNA-seq have provided ways to further identify and characterize cell-types at finer resolutions [14–18, 21], which was not possible using bulk RNA-seq. In addition, many scRNA-seq studies have investigated tumor microenvironment by immune cells as this has been shown to be an important target in HCC treatment in the era of immunotherapy, with potential prognostic utilities [22, 23]. The importance of understanding tumor heterogeneity is further illustrated by the clinical observation that NAFLD-related HCC cases may be more resistant to new systemic immunotherapies [24], as shown at the molecular level both in human studies and murine models [22]. Thus, given the changing landscape of HCC etiologies and the observed clinical heterogeneity, additional cell-type level transcriptomics studies of HCC are warranted.

We hypothesized that snRNA-seq can complement the existing scRNA and bulk expression data from liver HCC and normal liver cohorts and that these data can be integrated to identify currently unknown HCC-associated cell-types that affect survival when their proportions expand in the tumor tissue. To this end, we first used a liver snRNA-seq data set that we previously generated from HCC tumor and adjacent non-tumor liver biopsies from patients with NAFLD-related HCC [25], and then integrated these data with two existing liver scRNA-seq data sets, representing multiple etiologies of HCC and normal liver [7, 8]. Thus, we generated a powerful reference data set, comprising both viral and non-viral origin HCC, adjacent non-tumor, and normal liver samples at the single cell resolution. We then leveraged the cell-type marker genes identified in these three reference data sets to decompose cell-type proportions in liver bulk RNA-seq data from the well-phenotyped Cancer Genome Atlas (TCGA) cohort [26] (361 HCC tumor and 49 adjacent non-tumor biopsies) to first accurately estimate the tumor/non-tumor cell-type proportions and then test the effects of the identified HCC-enriched cell-types on survival outcomes. To replicate and further validate the results, we used the Liver Cancer Institute (LCI) cohort [27] (221 HCCs and 209 adjacent non-tumor tissue biopsies), collected by the Liver Cancer Institute and Zhongshan Hospital of Fudan University, which consists predominantly of chronic hepatitis B-HCCs. Using these two independent HCC cohorts, we discovered a replicated, proliferative cell-type, Prol, characterized by 656 mitosis and cell-cycle enriched cell-type marker genes, that is significantly more present in the HCC cases than in adjacent non-tumor liver tissue both in TCGA and LCI, in line with our single cell level data. Previous studies have not identified HCC cell-types associated with survival. Thus, our discovery that HCCs with a high Prol cell-type content have significantly worse survival outcomes advances the field by elucidating a key HCC risk cell-type. Importantly, we observed this same result both in TCGA and LCI, which increases the scientific rigor of our finding.

Multiple cancer genes and mutations have been identified in HCC, including mutations in tumor suppressors, such as tumor protein P53 (*TP53*) [6]. However, it is not known whether these somatic mutations are also associated with cell-type changes in HCC. To address this knowledge gap and elucidate the molecular mechanisms of the identified cell-types, we investigated the HCC risk cell-type, Prol, for accumulation of known somatic cancer mutations [6, 28]. Using somatic mutation of origin analysis, we discovered that somatic *TP53* and *RB1* mutations are linked to the identified increase of Prol in HCC.

## Methods

### Study design

To identify cell-types associated with HCC and its survival outcomes, we first analyzed three liver single cell level data sets from an existing snRNA-seq cohort of NAFLD-related HCC [25], an existing scRNA-seq cohort of HCC from various etiologies [8], and a healthy liver scRNA-seq cohort [7] to identify and characterize their cell-types. Next, we leveraged these liver cell-type reference data to decompose cell-type proportions in the liver bulk RNA-seq data from the Cancer Genome Atlas (TCGA) cohort [26] and subsequently tested the estimated cell-type proportions for associations with HCC and survival outcomes. Then, the HCC and survival associated cell-types identified in TCGA were tested for replication in independent liver bulk microarray expression data from the previously published LCI cohort [27]. Finally, we searched for associations between cell-type proportions and somatic mutations in the TCGA cohort.

### snRNA-seq cohort

We identified NAFLD-related HCC cases among patients undergoing surgical resection for HCC treatment at the Dumont-UCLA Liver Cancer Center [25]. The 3 patients with NAFLD-related HCC were women with a mean age of 77.9 ± 3.1 years and a mean body mass index of 25.3 ± 2.9 kg/m^2^, who had components of the metabolic syndrome (hypertension, dyslipidemia and insulin resistance). All patients exhibited features of nonalcoholic steatohepatitis (NASH) on liver histopathology (steatosis, ballooning and lobular inflammation [29]), and none had cirrhosis, as assessed by the METAVIR fibrosis score [30] (Additional file 1: Fig. S1). All patients also presented with clinically heterogeneous tumors, based on sizes, histological stages of differentiation (moderate to poorly differentiated), and serum alpha fetoprotein (AFP) levels, with one patient exhibiting an AFP of > 400 ng/mL.

Tissues were characterized by a pathologist using H&E and immunohistochemical stains, which confirmed the diagnoses of HCC (*n* = 3) and adjacent non-tumor (*n* = 3). Samples were snap frozen and kept at − 80^0^C until extraction of the nuclei. All histopathology slides were reviewed by the same pathologist. We abstracted clinical data and other demographics from the electronic health records. The study was approved by the UCLA IRB, and all participants provided a written informed consent.

### Two existing scRNA-seq cohorts

Along with the snRNA-seq data [25], we also incorporated liver scRNA-seq data from two previously published cohorts into our single cell level analysis [1]: HCC patients with viral origin of HCC (*n* = 4), HCC patients with unspecific origin of HCC (*n* = 10), and adjacent control liver samples (*n* = 14), as well as a healthy normal donor, collected in the Singapore General Hospital and National University Hospital [8], and [2] normal liver samples (*n* = 9), collected in the Strasbourg University Hospital [7]. Data from Sharma et al. [8] were downloaded from https://data.mendeley.com/datasets/6wmzcskt6k/1. Read counts for filtered droplets (*n* = 73,589) from the 14 HCC patients and 1 control were extracted from the downloaded HCC.h5ad file. Read counts for the 9 normal liver samples from Aizarani et al. [7] were downloaded from GEO under the accession number GSE124395. We used the filtered set of droplets provided by the authors (*n* = 10,372) for analysis.

### Processing of The Cancer Genome Atlas (TCGA) bulk RNA-seq, mutation, and clinical data

To expand our cell-type composition analysis to a larger number of HCC samples, we leveraged data from The Cancer Genome Atlas Liver Hepatocellular Carcinoma (TCGA-LIHC) (referenced as TCGA in the text) [26]. The TCGA-LIHC cohort includes 361 cases with primary tumors. We included only those cases that were designated as non-recurrent primary HCC and excluded cholangiocarcinomas, HCC-cholangiocarcinoma mixed tumors, and other rarer types of HCC, such as fibrolamellar, as these have different pathogenesis and clinical outcomes. We integrated bulk RNA-seq, mutation, clinical, and survival data with our single cell level RNA-seq data to identify HCC-associated cell-types.

Clinical data were downloaded from Genomics Data Commons (GDC) portal [31] (https://portal.gdc.cancer.gov/projects/TCGA-LIHC). We abstracted the available clinical and biospecimen data from Genomic Common Data portal, which included age, sex, ethnicity, and HCC tumor size, as well as node and metastatic American Joint Committee on Cancer (AJCC) TNM staging, and RNA integration number (RIN). Some other clinical characteristics were missing in TCGA, and thus, we had no data on cirrhosis status, the Model for End-Stage Liver Disease (MELD), serum AFP levels, or additional clinical phenotypes (e.g., diabetes and medication). Endpoint data for the survival analysis were downloaded from Table S1 from Liu et al. [32], and redacted cases were removed before analysis.

Liver bulk RNA-seq expression data were downloaded from the GDC portal [31] as HTSeq counts for all TCGA-LIHC individuals. We included counts for the 361 primary tumor samples, as well as for 49 matched non-tumor samples. For downstream analysis, the counts were Trimmed Mean of M-values (TMM) normalized with edgeR [33] and log10 transformed after adding a prior count of 1. Finally, RIN was regressed out to obtain the final normalized expression data.

Somatic mutation data collected from whole exome sequencing of tumor biopsies for the TCGA-LIHC were downloaded from the Broad Genome Data Analysis Center (GDAC) (http://gdac.broadinstitute.org). The Analysis Results file from the MutSig2CV under Mutation Analyses were downloaded on May 18, 2021. These included a MAF file of somatic mutations for each sample (LIHC-TP.final_analysis_set.maf), as well as a list of 69 significantly frequently mutated HCC genes (*q* < 0.1) (sig_genes.txt).

### The Liver Cancer Institute (LCI) cohort used for replication analyses

To validate the results obtained in TCGA, we analyzed a previously published HCC microarray dataset [27, 34]. This study recruited the HCC patients from the Liver Cancer Institute (LCI) and Zhongshan Hospital of Fudan University, most of whom had a history of chronic hepatitis B (HBV) infection. We obtained tumor microarray expression, clinical, and overall survival (OS) outcome data for a total of 221 patients. Additionally, 209 of these patients had expression data for adjacent non-tumor liver biopsies. RMA-normalized microarray expression data in log space were directly downloaded from GSE14520 in GEO. The clinical data, including OS endpoints, were downloaded as the extra endpoint text file from GSE14520. The expression data had been normalized by the authors [27], and thus, we used them directly for downstream analysis.

### Liver single nucleus extraction for snRNA-seq

For the snRNA-seq of the 3 NAFLD-related HCC and 3 adjacent non-tumor control biopsies, we cut the frozen samples over dry ice and placed them in glass tubes, as described earlier [25]. Briefly, we added 4 ml of lysis buffer consisting of 0.1% IGEPAL, 10 mM Tris-HCl, 10 mM NaCl, and 3 mM MgCl2 to the tissue. After 10 min on ice, we mechanically homogenized the tissue using a Dounce homogenizer, and then filtered them through a 70-μm MACS smart strainer (Miltenyi Biotec #130-098-462) to remove debris. We isolated the nuclei by spinning the homogenate at 500 x g for 5 minutes at 4 °C and washed the nuclei in 1 ml of resuspension buffer (RSB) consisting of 1X PBS, 1.0% BSA, and 0.2 U/μl RNase inhibitor. We filtered the nuclei a second time using 40 μm Flowmi cell strainer (Sigma Aldrich # BAH136800040) and centrifuged them at 500×*g* for 5 min at 4 °C. We resuspended the nuclei in the wash buffer and kept them on ice. To assess nuclei isolation (for clumping and intact membrane), we labeled the nuclei with Hoechst stain and counted them using BZ-X710 fluorescent microscope. Nuclei were immediately processed them with the 10X Chromium platform following the Single Cell 3′ v2 protocol. We generated libraries with the 10X platform and sequenced the nuclei on an Illumina NovaSeq S2 at a sequencing depth of 300–400 million reads per sample.

### Processing of the snRNA-seq data

Before read alignment, we trimmed template switch oligos, primers, and polyA sequences greater than 20 base pairs from the fastq reads using cutadapt (https://cutadapt.readthedocs.io/en/stable/). We aligned reads to the GRCh38 human genome reference and Gencode v26 [35] gene annotations using STARSolo in STAR v2.7.3a [36]. Gene counts were taken from the full pre-mRNA transcript using the “—soloFeatures GeneFull” option. We filtered empty and contaminated droplets using Debris Identification using Expectation Maximization (DIEM) [13], where we further adapted estimation of the multinomial mixture model parameters by adding a prior count of 1 to the gene mean estimates and the cluster membership estimates to avoid overfitting. To further remove doublets and contaminated clusters from the snRNA-seq data, we separately clustered parenchymal hepatocytes and non-parenchymal nuclei. Nuclei were clustered in a first pass and assigned to hepatocyte and non-hepatocyte cell-types. Each group was clustered again separately. Then, we removed nuclei belonging to clusters expressing markers from multiple cell-types, leaving the filtered set of nuclei (*n* = 39,995).

### Integration and clustering of the single cell level data from the three cohorts

To analyze the single-cell level data across the cohorts, we first removed cohort- and experiment-specific effects by performing data integration. Counts were first normalized using sctransform [37] and integrated using canonical correlation analysis (CCA) [38]. Integrations were performed across the 6 NASH-HCC samples, the 15 patients (14 HCC and 1 healthy control) in the Sharma [8] data set, and the single combined set of 9 samples in the Aizarani [7] data set. The 22 samples across the 3 cohorts were used for independent samples during normalization and integration. Each of the 22 samples were normalized with sctransform using 3000 genes for the number of variable features. To reduce the time required for integration, we selected a subset of the 22 samples for use as a reference during the FindIntegrationAnchors step. We selected 11 samples, including the combined sample from Aizarani et al. [7] to serve as a healthy control, and 10 additional randomly selected samples. After anchor identification, all 22 samples from the 3 cohorts were integrated with the IntegrateData function in Seurat [38] using 30 dimensions. This resulted in corrected counts for the 123,956 droplets. Finally, we performed clustering on these corrected counts for downstream cell-type assignment. We ran principal component analysis (PCA) and constructed the shared nearest neighbor (SNN) graph with 30 PCs. This graph was used as the input to Louvain clustering by running the FindClusters function with a resolution of 1 [38]. We chose a resolution of 1 to accommodate the large number of cells and nuclei and better identify subtypes. To evaluate the effect of integration, we also clustered cells and nuclei in the three cohorts by clustering the merged data without CCA integration. Sctransform was run on the merged counts as described above, treating the cells and nuclei from the three cohorts as a single sample. PCA and clustering were performed on the sctransformed counts in the same manner as for the integrated data.

### Marker gene identification and cell-type assignment of single cell level data

For cell-type classification, we obtained the upregulated marker genes and log-fold changes for each cluster using the uncorrected, log-normalized counts. Raw counts for all droplets were multiplied by a scaling factor to sum to 1,000 as this was the approximate median across all droplets, and then log-transformed. To identify marker genes, we performed a logistic regression test using the FindAllMarkers function in Seurat [38] and kept marker genes with an average log_2_ fold change of at least 0.1 and Bonferroni-adjusted *p*-value < 0.05 corrected for the total number of genes in the data set. For the pathway enrichment analysis, we also obtained the log fold changes for all expressed genes by calculating the difference in log_2_ means between the counts of droplets classified within and outside of the cluster. Cell-types were assigned based on manual curation of known marker genes [26]. Throughout the manuscript, we call the 25 assigned clusters the subcell-types. We further merged the subcell-types into the 8 main cell-types based on their common lineage, expressed genes, and enriched pathways.

### Pathway enrichment analyses of the single cell level data

To gain insight into cell-type functions in the liver single cell level data, we performed pathway enrichment analysis of upregulated marker genes for each liver subcell-type. We used the clusterProfiler [39] R package to run gene set enrichment analysis (GSEA) [40]. We tested for enrichments of the pathways in the Reactome database [41, 42]. For each subcell-type, its log fold changes were used to rank the gene set as input to the gsePathway function, using 10,000 permutations and an epsilon of 1 × 10^−50^. *p*-values were corrected for multiple testing using FDR.

### Clustering of Prol cells and nuclei

The Prol cluster that we identified in the integrated analysis expressed markers involved in cell division; however, our integrated analysis did not further separate these cells/nuclei, so we subclustered the 1,743 Prol cells/nuclei to identify its composition. We ran a clustering pipeline similar to the whole data set, with modifications to account for the lower number of cells. The Prol cells/nuclei were first split by cohort, and sctransform was run on the raw counts for each of the three samples. We then ran CCA integration with the k.filter and k.weight parameters set to 75 to account for the small number of cells/nuclei in each cohort, as only 92 Prol cells were present in the healthy liver tissues from the Aizarani data set [7]. Cluster assignments and UMAPs were generated using 30 PCs with a resolution of 0.2 to accommodate the lower number of cells/nuclei and to match clusters with the main cell-types.

To assign Prol cells/nuclei to the main liver cell-types, we used SingleR [43]. For classification, we first generated a reference of the 7 main cell-types (excluding the Prol cluster) from the integrated liver data. Briefly, pairwise T-tests were performed across the 7 main cell-types and the top 100 markers were extracted. A reference was derived on the log-normalized counts using these top markers with the trainSingleR function. To account for the single-cell level nature of the reference, the counts were aggregated to pseudobulk samples with the aggr.ref parameter. Finally, we ran the classifySingleR function on the droplets in the Prol cluster and assigned their cell-type to the pruned labels.

### Estimating cell-type proportions and correlation analyses of the cell-type marker genes in the liver bulk RNA-seq from TCGA and microarray data from LCI

To estimate cell-type proportions in the bulk liver expression data in the TCGA-LIHC cohort [26], we used a co-expression based approach implemented in Bisque [14]. Briefly, this approach performs PCA on the top cell-type marker genes for each cell-type. We used normalized RNA-seq expression and cell-type markers as input, requiring a minimum of 20 genes and a maximum of 300 genes for the set of markers for PCA. The marker genes were obtained from our single cell level reference data. Decomposition was performed for the 8 main cell-types and 25 subcell-types. As we observed high correlation (*R* > 0.9) between proportion estimates of subcell-types within their main classification, we used only the proportion estimates for the main cell-types for downstream analysis.

In order to replicate our results with the decomposed proportion estimates observed in TCGA, we ran the same decomposition in the LCI cohort. We ran Bisque on the normalized microarray expression data using the same parameters and marker gene input described above. To assess the reliability of the TCGA and LCI proportion estimates, we analyzed the co-expression patterns of the marker genes in each cohort. We found that for the LCI cohort, the B cell marker genes did not show positive correlations across their expression. As our decomposition approach relies on co-expression of marker genes, we excluded B cells from the LCI main cell-type proportion estimates.

### Cell-type proportion differences between tumor and non-tumor in the single cell level and bulk data

To identify tumor-enriched or depleted cell-types, we performed paired Wilcoxon signed-rank tests between tumor and non-tumor samples. In the single-cell-level data, we calculated differences in the observed proportions between paired tumor and non-tumor samples in the 17 patients with matched biopsies. Differences were calculated for each subcell-type. The observed subcell-type proportions for each tumor or non-tumor sample were calculated by dividing the number of cells/nuclei in the subcell-type by the total number in the sample. For the tumor samples in the Sharma data set [8], the core and peripheral tumor droplets were combined. *p*-values were corrected for testing 25 subcell-types using FDR.

For calculating differences in cell-type proportion estimates between tumor and adjacent non-tumor samples in the TCGA and LCI bulk tissue cohorts, we performed a paired Wilcoxon test in TCGA (*n* = 49) and LCI (*n* = 209). *p*-values were corrected for testing 8 and 7 cell-types in the TCGA and LCI cohorts, respectively, using FDR.

### Survival outcome associations with cell-type proportion estimates

To investigate the effect of cell-types on survival outcomes, we performed associations between survival outcomes and cell-type proportion estimates. Associations were carried out with Cox proportional hazard regressions for overall survival (OS) and progression free interval (PFI) in TCGA, and OS in the LCI validation cohort. We included age, sex, and ethnicity as covariates in TCGA, and age and sex in the LCI cohort, as most patients from this cohort were of Asian descent. In addition, we included tumor stage as a binary covariate where specified, where patients with stage I and II were grouped into the low group and those with stage III and IV were grouped into the high group. Patients with any missing covariate data were excluded. All *p*-values were corrected for multiple testing using false discovery rate (FDR). All survival analyses were performed with the survival package in R [44]. We tested survival differences between low vs. high proportion groups, splitting the participants by median or quartile. In the median analysis, tumor samples with proportion estimates below and above the median were grouped into low and high, respectively. Similarly, the quartile analysis was performed using the lower and upper 25% quartiles of the cell-type proportion estimates as cutoffs. Plots were generated using the Kaplan-Meier method without any covariates. Unless otherwise specified, all cell-type effects were corrected for testing 8 and 7 cell-types in the TCGA and LCI cohorts, respectively, using FDR.

### Mutation analyses in TCGA-LIHC

We hypothesized that mutations in distinct genes would lead to increased Prol proportions in HCC tumor samples. We thus tested for differences in proportions between tumor samples with and without a somatic mutation in TCGA-LIHC, as LCI did not profile tumor mutations. Somatic mutations in TCGA-LIHC were collected from exome sequencing data processed by GDAC (http://gdac.broadinstitute.org). We restricted our analysis to 69 genes frequently and significantly mutated in HCC, as reported previously in the TCGA-LIHC cohort (http://gdac.broadinstitute.org) [45]. A gene was considered significantly mutated if its *q*-value was less than 0.1, as determined by MutSig2CV [46]. Tumor samples with at least one synonymous, nonsense, in frame, splice site, missense, or frame shift variant were considered as having a somatic mutation (mut.). Tumor samples without any somatic mutation detected were considered as wildtype (WT). For each gene and each main cell-type, we used a Wilcoxon test to assess the difference in cell-type proportion estimates between tumor samples with a somatic mutation detected and tumor samples without a somatic mutation. For *TP53*, we also tested for tumor proportion differences between wildtype (WT) cases and each of the somatic mutation types listed previously. Wilcoxon *p*-values were adjusted for multiple testing across all gene-main-cell-type pairs using FDR.

### Bulk liver differential expression (DE) analyses

In addition to estimating cell-type proportions, we also reciprocally evaluated the significance of cell-types in HCC by assessing single cell expression of genome-wide significant bulk tumor-elevated, survival decreasing, and mutation-elevated genes. To first obtain the genome-wide tumor-elevated genes in TCGA and LCI, we ran differential expression (DE) genome-wide in both the TCGA and LCI bulk expression cohorts. DE was run on the 49 and 209 paired samples in the TCGA and LCI, respectively, that contained the matched tumor and adjacent non-tumor samples. For the TCGA RNA-seq data, we first filtered for expressed genes by removing those with an average number of reads less than 10 across the 98 samples. We then ran edgeR [33] on the TMM normalized counts with the generalized linear model (GLM) framework (glmFit and glmLRT functions) and setting a prior count of 1. For the LCI microarray data, we used the gene-filtered and normalized data provided. We then ran limma [47] to fit a linear model (lmFit function) and compute test statistics with empirical Bayes shrinkage of variances (eBayes function). For both the TCGA and LCI, we accounted for the paired status of the samples by including the patient as an indicator covariate.

Next, to obtain the genome-wide survival-decreasing genes in the bulk expression cohorts, i.e., OS- and PFI-decreasing genes in TCGA and OS-decreasing genes in LCI, we performed Cox proportional hazard regressions for OS and PFI in TCGA, and OS in the LCI validation cohort. As with the proportion analyses, we included age, sex, and ethnicity as covariates in TCGA, and age and sex in the LCI cohort. Patients with any missing covariate data were excluded. We then ran Cox proportional hazards regression testing normalized gene expression values as a quantitative predictor against survival outcomes. The statistical significance of these survival-decreasing genes was corrected for genome-wide testing using FDR. Regressions were performed with the survival package in R [44].

Similarly, to identify genes upregulated in the context of a somatic mutation in *TP53* and *RB1*, we also performed genome-wide DE between somatic mutation (mut.) and wildtype (WT) carriers in the TCGA cohort. We broadly included genes with greater than 0 counts in at least 50% of the 410 samples. To test for DE, we ran the GLM framework in edgeR [33] using TMM normalization. DE was run on the 357 primary tumor samples with both mutation and RNA-seq data. We tested for differences in bulk liver expression between participants that were wildtype (WT) and those that had a somatic mutation (mut.) in the particular gene. A genome-wide DE analysis was performed for both *TP53* and *RB1*.

### Scoring of the cell-cycle, tumor-elevated, OS- and PFI-decreasing, and mutation upregulated bulk genes in the single cell level data

To assess cell/nuclei expression of the cell-cycle genes [48] (42 S phase genes and 54 G2 and M phase genes) as well as the tumor-elevated, OS- and PFI-decreasing, and mutation upregulated genes identified in our bulk DE analyses (see above), we assigned module scores with the AddModuleScore function implemented in the Seurat package [38]. Briefly, module scores are derived by calculating the average expression of the gene set and subtracting the average expression of gene sets. Control gene sets are randomly selected from bins based on average expression. The expression data of cells/nuclei for module scoring were calculated by multiplying raw read counts to sum to 1,000 and log transforming them.

For cell cycle scoring, the gene sets included 42 S phase genes and 54 G2 and M phase genes provided in the Seurat package [38, 48]. For tumor-elevated scores, we used the genes identified in the bulk liver DE analysis that had a log fold change greater than 1 of tumor over non-tumor and an FDR-corrected *p*-value < 0.05. The tumor-elevated gene set included 1065 genes in TCGA and 335 genes in LCI. For the OS- and PFI-decreasing gene sets, we analyzed the genes identified in the genome-wide survival analysis of the bulk liver data that had a hazard ratio > 1 (increased expression leading to a worse prognosis) and an FDR-corrected *p*-value < 0.05. There were 740 OS-decreasing genes and 528 PFI-decreasing genes in TCGA and 36 OS-decreasing genes in LCI. For the mutation upregulated genes, we included those from the genome-wide DE mutation analysis for *TP53* and *RB1* that had a log fold change greater than 0.5 and an FDR adjusted *p*-value < 0.05. This resulted in a set of 1358 *TP53* mut. upregulated genes and a set of 774 *RB1* mut. upregulated genes.

Differences in tumor-elevated, OS-decreasing, PFI-decreasing, and mutation upregulated gene scores between the Prol and all other clusters were assessed by running a Wilcoxon test between droplet scores within and outside of the Prol cluster.

## Results

### Overview of study design

HCCs are poorly characterized at the cell-type level. To address this scientific and biomedical knowledge gap, we utilized the following three single cell level RNA-seq data sets to produce a comprehensive cell-type reference for HCC tumor, adjacent non-tumor tissue, and normal livers [1]: liver snRNA-seq data that we previously generated from HCC samples (*n* = 3) and adjacent non-tumor control tissue samples (*n* = 3) from patients with NAFLD-related HCC undergoing hepatic resection [25] [2]; existing liver scRNA-seq data from HCC patients with viral origin of HCC (*n* = 4), HCC patients with unknown etiology of origin of HCC (*n* = 10), adjacent non-tumor control tissue samples (*n* = 14), and a healthy control liver sample (*n* = 1) [8]; and [3] existing liver scRNA-seq data from normal liver samples (*n* = 9) [7]. After integrating these data and identifying the cell-types, we leveraged the cell-type transcriptional profiles to estimate cell-type proportions (decompose) in bulk liver RNA-seq samples from the well-established TCGA cohort [26] (361 patients with primary HCC tumors, 49 of whom have paired adjacent non-tumor tissue samples) and searched for cell-types that are significantly enriched in HCC. To replicate these findings, we used the LCI cohort [27] with microarray data from 221 patients with primary HCC tumors, of whom 209 have paired adjacent non-tumor biopsies. Next, we tested the effect of the HCC-increased cell-type on survival outcomes in the TCGA and LCI cohorts. Finally, we searched for associations between somatic mutations and the increased cell-type proportion estimates in HCC (for the overall study design, see Additional file 1: Fig. S2).

### Data integration, clustering, and cell-type assignment in three single cell level RNA-seq cohorts

**Fig. 1:**
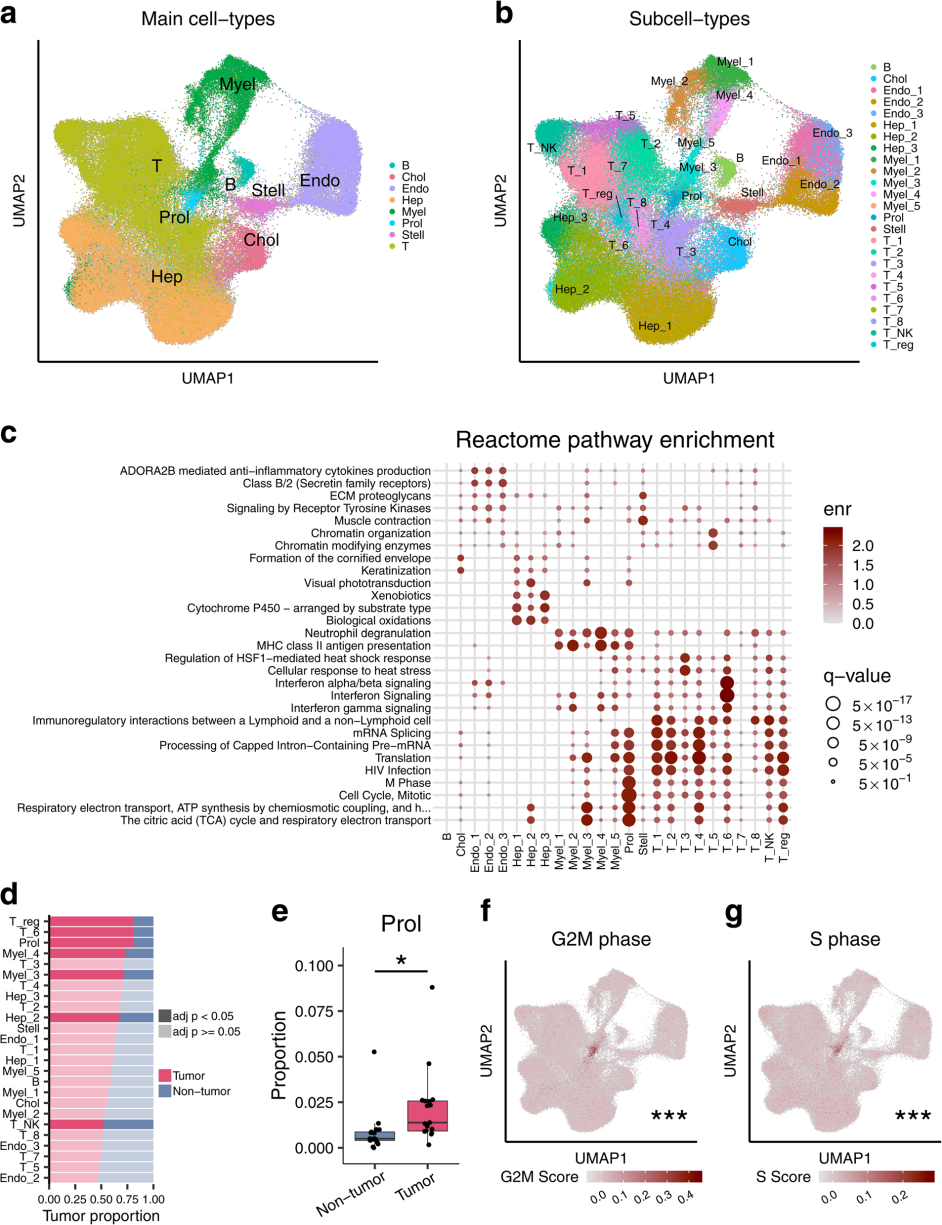
Multi-cohort integration of three liver HCC single cell level data sets identifies and characterizes an HCC-associated cell-type. We assessed liver cell-types and HCC-related cell-type changes by integrating Aizarani et al. [7] scRNA-seq data (*n* = 9 non-HCC samples), Sharma et al. [8] scRNA-seq data (*n* = 1 non-HCC, *n* = 14 HCC-tumor, and *n* = 14 adjacent non-tumor samples), and Rao et al. [25] snRNA-seq data (*n* = 3 HCC-tumor and *n* = 3 non-tumor samples). **a**, **b** Uniform Manifold Approximation and Projection (UMAP) visualization of 123,956 cells and nuclei integrated to remove cohort-specific effects. Clusters were assigned to (**a)** 8 major cell-types and (**b)** 25 subcell-types. **c** Pathway gene set enrichment analysis of the expression profiles for each subcell-type using the Reactome pathway database. The enr values indicate normalized enrichment scores and *q*-values denote Benjamini-Hochberg-adjusted *p*-values. Full pathway names are shown in Additional file 3: Table S2. **d** The bar plot shows the proportion of cells/nuclei in the full set of 123,956 cells/nuclei originating from HCC tumor and non-tumor samples separated by subcell-type. Darker fills indicate an FDR-adjusted *p*-value < 0.05 from a paired Wilcoxon test between proportions of HCC tumor and non-tumor samples. **e** Proportions of the Proliferative (Prol) cell-type are significantly higher in the 17 HCC tumor samples than in their 17 adjacent paired non-tumor samples after correcting for multiple testing with FDR, as assessed by a paired Wilcoxon test. **f**, **g** UMAP plots with cells/nuclei colored by their cell cycle score in the full single-cell level RNA-seq data of 123,956 droplets show that the Prol cluster consists of droplets with higher expression of (**f)** G2M phase genes and (**g)** S phase genes. The asterisks denote the significance of a difference between G2M and S phase gene scores between Prol and non-Prol cells/nuclei. Significance levels for *p*-values in (**e**–**g)** *p < 0.05, **p < 0.005, ***p < 0.0005. B indicates B cells; Chol, cholangiocytes; Endo, endothelial cells; Hep, hepatocytes; Myel, myeloid cells; Stell, stellate cells; and T, T cells

To decompose liver bulk RNA-seq cell-types in the TCGA and LCI cohorts, we first set up a single cell level reference data set. We utilized three single cell level cohorts generated using either snRNA-seq or scRNA-seq to build a powerful liver cell-type reference data set with a large number of cells and multiple HCC etiologies represented. Briefly, the included cohorts consist of both viral and non-viral origin HCC biopsy samples, adjacent non-tumor control samples, and normal liver samples (for cohort descriptions see Methods). Merging of the three data sets without integration resulted in cohort-specific clustering, indicating the presence of batch effects (Additional file 1: Fig. S3). When merging without integration, we also observed evidence of inter-patient heterogeneity across the 17 paired HCC samples (Additional file 1: Fig. S3). In order to identify shared cell-types and correct for these batch effects, we integrated these single cell level expression data using the CCA approach [38, 49] that should retain biologically meaningful signals while reducing technical variance (Fig. 1a,b). The integrated data were clustered using Seurat [38], resulting in the identification of 25 cell-types (Fig. 1a,b).

### Discovery of an HCC-associated, cell-cycle-related cell-type in the single cell level data

Clustering of the integrated single cell level data (123,956 analyzed nuclei/cells) identified 25 subcell-types in total (Fig. 1b), which we merged and classified into 8 main cell-types based on their lineage (Fig. 1a). Subcell-types and main-cell types were classified based on expression of known marker genes and enriched pathways (Fig. 1c; Additional file 1: Fig. S4; Additional file 2: Table S1 and Additional file 3: Table S2). We then searched for subcell-types/cell-types enriched or depleted in HCC tumor cells (Fig. 1d). We observed a significant enrichment of tumor cells (80.4%) in a new cell-type cluster that we named Proliferative (Prol) cell-type (Fig. 1d,e). The pathway analysis of its marker genes suggested that this tumor-enriched cell-type consists of mitotic cells (Fig. 1c, see below). We also observed a significantly increased number of tumor cells in T, myeloid, and hepatocyte subcell-types and a decreased number of tumor cells in natural killer T subcell-type (Fig. 1d). Thus, our multi-cohort integration of both snRNA-seq and scRNA-seq data allowed us to identify the tumor cell-enriched Prol cell-type that had not been identified previously. The top pathway enrichments of the Prol marker genes were oxidative phosphorylation and cell cycle, suggesting that their functions are related to growth and cell division (Fig. 1c). To further investigate the proliferative capacity of Prol, we assigned G2M and S module scores based on average expression of G2M and S cell cycle genes [48] (see Methods), respectively. We found that cells/nuclei from Prol demonstrated significantly higher S and G2M phase module scores when compared to other cell-types (Fig. 1f,g). The higher cell cycle scores imply that Prol consists of actively dividing cells. To determine the cell-type composition of these proliferating cells, we re-classified the Prol cells/nuclei into main cell-types using a reference trained on the non-Prol cells/nuclei. In addition to hepatocytes, all non-parenchymal cell-types were observed in this tumor-enriched cluster (Additional file 1: Fig. S5). This presence of dividing non-hepatocyte cells observed in the tumor-enriched Prol cluster highlights the importance of the microenvironment in supporting HCC growth [50].

We next explored the marker genes within the Prol cell-type to further understand its biology. We identified 656 protein-coding marker genes in Prol, of which 15 had a log fold change > 1 for differential expression between the Prol and other cell-types (Additional file 2: Table S2). Most of these 15 strongest Prol marker genes (12/15; 80%) had previously been identified in HCC pathogenesis or associated with clinical features of the disease [51–58]. Consistent with our findings, liver bulk expression of the histone protein, *H2AFZ*, a marker gene in Prol, was also identified in an independent HCC study to be associated with cell cycle genes regulated by TP53 [59]. However, among the 15, we discovered three genes, *HMGN2*, *RARRES2*, and *HIST1H4C*, which have previously been described in other malignancies [60, 61] but not in HCC. Two of these, *HIST1H4C* and *HMGN2*, are nuclear proteins that bind to nucleosomal DNA, consistent with Prol having higher S and G2M scores (Fig. 1f, g).

Overall, the single cell level reference data suggest that the Prol cell-type is associated with HCC. Therefore, we next used this single cell level reference data set to decompose cell-type proportions in the liver bulk RNA-seq HCC cohorts, TCGA and LCI, and then tested them for cell-type proportion differences between the HCC tumor and adjacent non-tumor control tissues.

### Decomposition of cell-type proportions in HCC and adjacent non-tumor samples discovers high proportions of the proliferative cell-type Prol in HCC

**Fig. 2:**
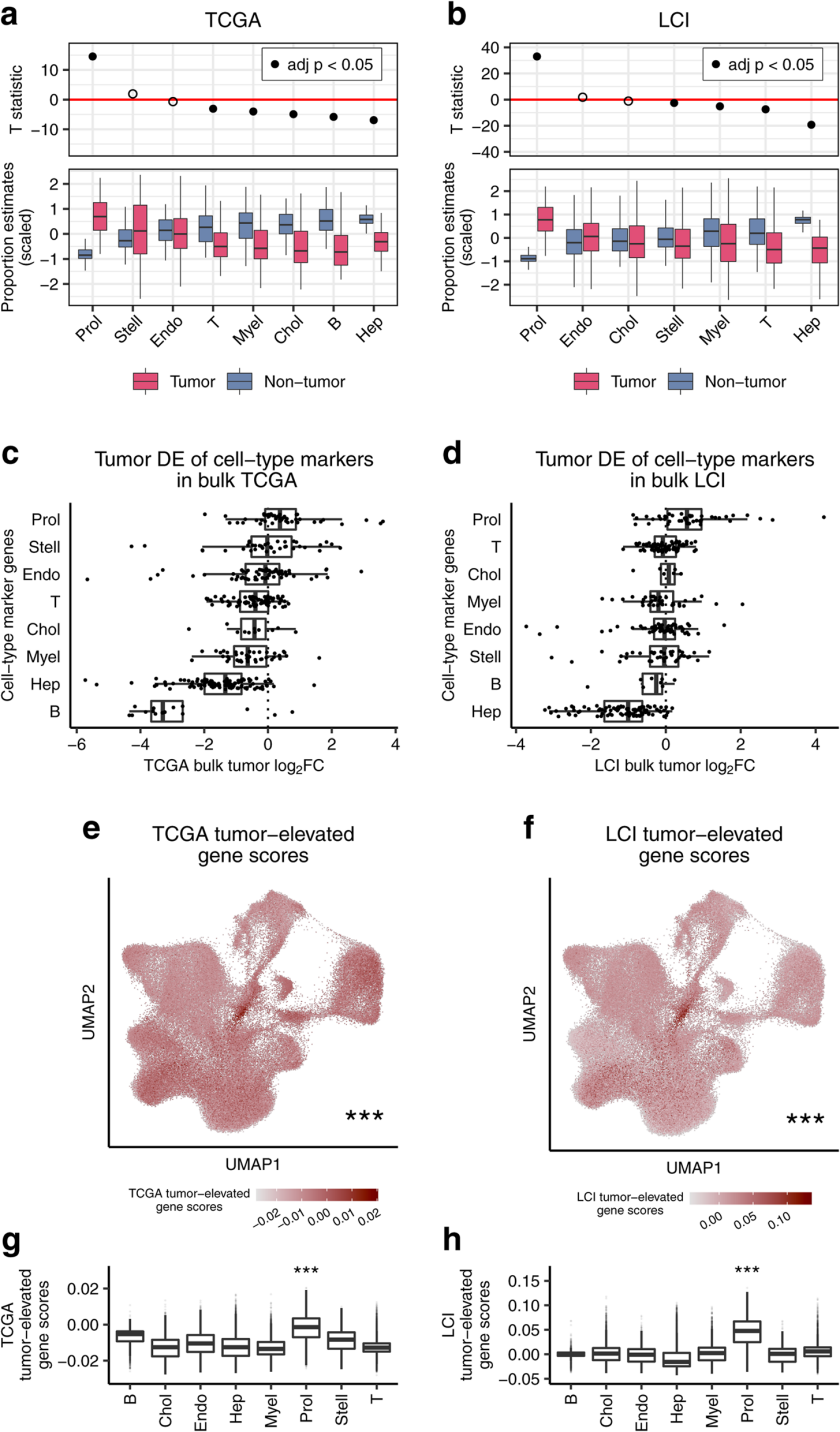
Among all cell-types decomposed in the TCGA and LCI bulk liver cohorts, Prol has the highest enrichment in HCC when compared to adjacent non-tumor tissue. The Prol cell-type shows consistent upregulation in HCC tumors in two independent liver bulk cohorts. **a**, **b** Proportions were estimated in the liver bulk RNA-seq data for the major cell-types identified in the single-cell level data and then tested for differential abundance between the tumor and non-tumor samples. The upper panel shows the T-statistic from a paired *t*-test between tumor and adjacent non-tumor tissue, with FDR-adjusted *p*-values calculated from a paired Wilcoxon test. The bottom panel shows a bar plot of the proportion estimates separated by tumor status. The differential abundance tests highlight the Prol cell-type as upregulated in the (**a)** TCGA (*n* = 49) and (**b)** LCI (*n* = 209) cohorts. B cell proportions were not estimated for LCI (**b**) as its marker genes did not show evidence of co-expression. **c**, **d** Association of the Prol cell-type with HCC tumors is highlighted by the log_2_ fold-changes (log_2_FC) of tumor over adjacent non-tumor samples for the marker genes of the cell-types that are indicated on the y-axis. Log_2_FC values were derived from a paired differential expression (DE) analysis in (**c)** TCGA (*n* = 49) and (**d)** LCI (*n* = 209) cohorts. **e**–**h** The Prol cells/nuclei significantly express tumor-elevated genes, as shown by droplet scores in the single-cell level data for tumor-elevated genes derived from the TCGA and LCI cohorts. Genome-wide DE analysis was performed between the paired tumor and non-tumor samples, and genes with an FDR-adjusted *p*-value less than 0.05 and a log_2_FC greater than 1 were considered tumor-elevated genes. Module scores of the tumor-elevated genes for each droplet were calculated based on their expression compared to a background set. **e**, **f** UMAP plots for (**e)** TCGA and (**f)** LCI are shown with cells and nuclei colored by their tumor module score. **g**, **h** Bar plots show the droplet tumor scores calculated from (**g)** TCGA and (**h)** LCI tumor-elevated genes separated by major cell-type. **e**–**h** Asterisks denote a significant difference in gene scores between Prol and non-Prol cells/nuclei as assessed by a Wilcoxon test. Significance levels for *p*-values: *p < 0.05, **p < 0.005, ***p < 0.0005

Next, we sought to determine whether cell-type composition changes observed in our single cell level reference data were conserved and universally present in HCCs. Therefore, we estimated cell-type proportions for the 8 main cell-types and 25 subcell-types from bulk liver RNA-seq data in the TCGA Liver Hepatocellular Carcinoma (TCGA-LIHC) cohort, consisting of 361 non-recurrent primary tumors and 49 paired adjacent non-tumor samples (total *n* = 410). We investigated the proportion estimates only for the 8 main cell-types as we found that estimates of the 25 subcell-types showed high intra-group correlation within their broader classifications (Additional file 1: Fig. S6), and these types of high correlations typically prevent accurate decomposition of subcell-types in bulk tissues [14]. For cell-type decomposition, we utilized Bisque [14], as described in detail in the Methods, resulting in proportion estimates for the 8 main cell-types. The marker genes of these main cell-types used for decomposition in Bisque (Additional file 4: Table S3) show high intra-cell-type co-expression and correlation with their respective proportion estimates (Additional file 1: Fig. S7a), suggesting their validity for estimating proportions. We then searched for differences in the abundance of these 8 cell-types between the paired HCC tumor and non-tumor tissue in TCGA. Of the 8 cell-types, we found that only Prol was significantly increased (Wilcoxon adjusted *p* = 5.68 × 10^−14^) in the 49 HCC tumors when compared to the paired adjacent non-tumor samples in TCGA, while 5 cell-types significantly decreased in tumors (Fig. 2a and Additional file 5: Table S4). This increase in Prol abundance was consistent with our observations in the single cell level data (Fig. 2a and Fig. 1d).

To replicate the cell-type differences we identified in the TCGA cohort, we investigated the LCI cohort that consists of mainly Asian HCC patients with HBV-HCC. We estimated the proportions of 7 of the 8 main cell-types in the liver microarray data from tumor (*n* = 221) and adjacent non-tumor (*n* = 209) biopsies (see Methods). We excluded B cells, as its marker genes showed little to no co-expression in the microarray data of this cohort, and thus the proportions could not be estimated reliably (Additional file 1: Fig. S7b). All of the other 7 main cell-types demonstrated higher intra-cell-type co-expression and correlations with their respective proportion estimates (Additional file 1: Fig. S7b). Then, we tested for differences between the tumor and adjacent non-tumor biopsies. We found strikingly similar cell-type changes between the tumor and non-tumor tissues in the LCI and TCGA cohorts (Fig. 2b and Additional file 5: Table S4). Only the Prol cell-type was significantly increased in HCC in the LCI cohort (Fig. 2b), while the myeloid, T, and Hep clusters were significantly decreased in both TCGA and LCI, with Hep showing the largest decrease (Fig. 2b). These replicated results show that Prol is the only consistently upregulated cell-type in HCC tumors using both the TCGA and LCI cohorts.

We then sought to validate the observed increase in the Prol proportion estimates in HCC tumors by analyzing gene-level differential expression between tumors and adjacent non-tumors from the bulk liver data. We first took the most specific cell-type marker genes with a log fold change > 0.5 in the single-cell level data and searched for differences in expression between the tumor and non-tumors in the bulk. The marker genes for the Prol cluster had the highest average log fold changes in both the TCGA and LCI cohorts when compared to all other cell types (Fig. 2c,d). We then performed a reciprocal analysis by taking all tumor upregulated genes with a log fold change greater than 1 in the bulk data and scoring cells/nuclei in the single-cell level data for their average expression using the module score option in Seurat [38]. We found that cells/nuclei from Prol had the highest bulk tumor scores when using the strongest tumor-upregulated genes from both TCGA (Wilcoxon *p* < 2.2 × 10^−16^) and LCI (Wilcoxon *p* < 2.2 × 10^−16^) (Fig. 2e–h). Taken together, the significantly increased expression of Prol marker genes at the bulk HCC tissue level, and vice versa the highest expression of the bulk tumor-upregulated genes in the Prol cell-type, support an increased abundance of the Prol cell-type itself in HCCs.

### The Prol cell-type is associated with HCC survival outcomes in TCGA and LCI

**Fig. 3:**
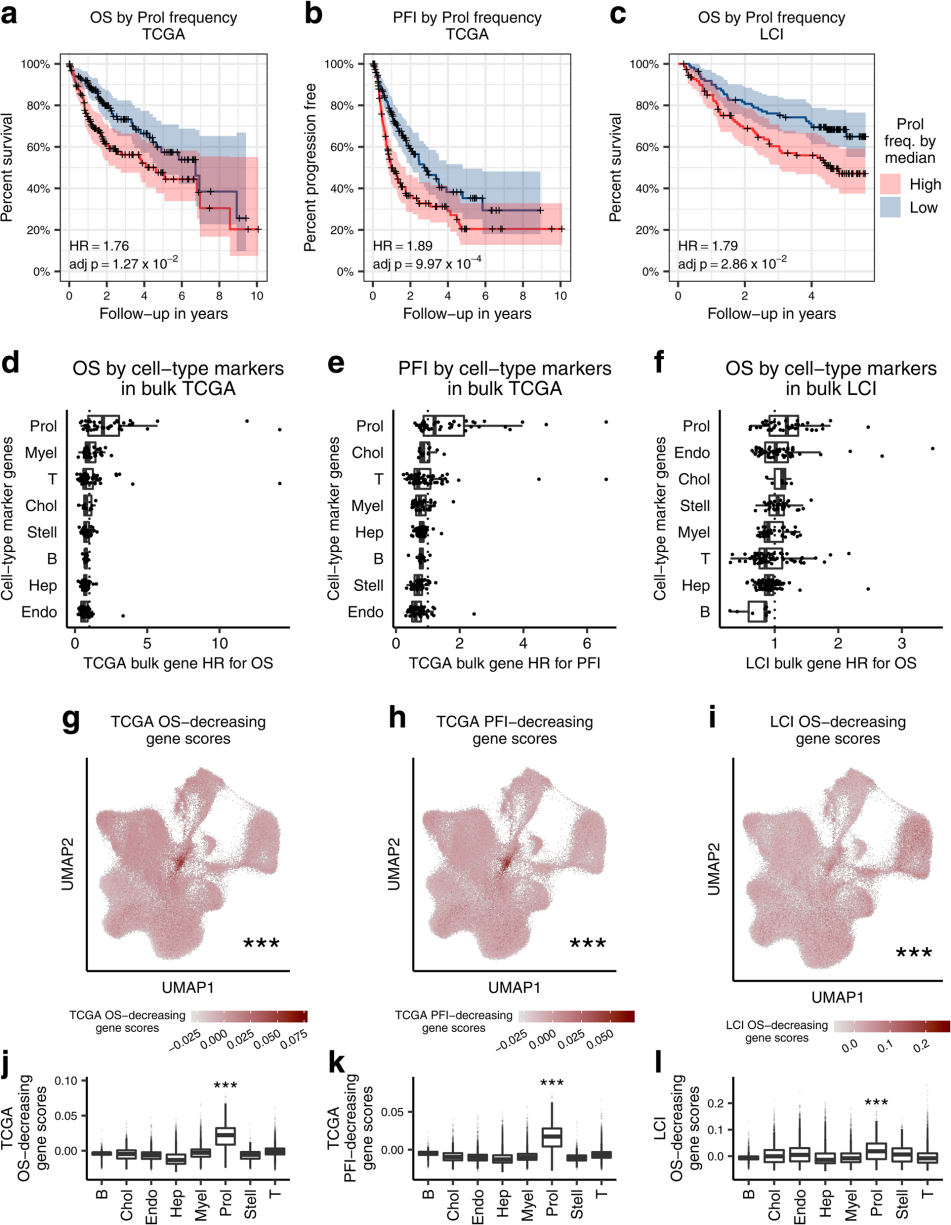
The HCC-enriched Prol cell-type associates with overall survival (OS) and progression free interval (PFI) in TCGA and with OS in LCI. Increased Prol cell-type proportion estimates are associated with poor survival outcomes in TCGA and LCI. **a**–**c** Kaplan-Meier survival curves for (**a)** overall survival (OS) and (**b)** progression free interval (PFI) in TCGA and (**c)** OS in LCI show worse survival outcomes for patients with high liver Prol cell-type frequency estimates. Patients with Prol frequency (freq.) estimates above and below the median were classified into high and low groups, respectively. The “+” signs on the line indicate right censoring of the event. The hazard ratios (HR) and FDR adjusted *p*-values were calculated from a Cox proportional hazards regression adjusting for age, sex, and for TCGA, race. **d**–**f** Association of the Prol cell-type with poor survival outcomes is highlighted by the HR values for cell-type marker genes calculated from a Cox proportional hazards regression of their expression in TCGA and LCI. Survival tests were performed for (**d)** OS and (**e)** PFI in TCGA and (**f)** OS in LCI. Each dot indicates a gene, with its HR on the *x*-axis and its cell-type on the *y*-axis. **g**–**l** Module scores of survival-decreasing genes in the single-cell level data are significantly higher in cells/nuclei from the Prol cell-type. Survival-decreasing genes were derived from genome-wide Cox proportional hazards regression analyses of all genes for the indicated event and cohort and taking the genes with FDR-adjusted *p*-values less than 0.05 and HR values greater than 1.0 into the module score analyses in (**g–l)**. **g**–**i** UMAP plots show cells/nuclei colored by (**g)** TCGA OS score, (**h)** TCGA PFI score, and (**i)** LCI OS scores. **j**, **l** Bar plots of survival-decreasing module scores for (**j)** TCGA OS, (**k)** TCGA PFI, and (**l)** LCI OS separated by the cell-type. **g**–**l** Asterisks denote a significant difference in survival-decreasing gene scores between Prol and non-Prol cells/nuclei as assessed by a Wilcoxon test. Significance levels for *p*-values: *p < 0.05, **p < 0.005, ***p < 0.0005

To determine the clinical significance of the Prol cell-type on survival outcomes in TCGA [32], we assessed its impact on overall survival (OS) and progression-free interval (PFI) in the 361 HCC patients. We hypothesized that an increased proportion of the tumor-associated Prol cell-type may be associated with poorer OS and PFI outcomes. To investigate this, we first associated the Prol cell-type proportions with survival outcomes in TCGA. We stratified the HCC patients into low and high cell-type proportion groups using the median (see Methods) and performed a Cox proportional hazards regression adjusting for age, sex, and ethnicity (Additional file 6: Table S5). Noteworthy, in TCGA, Prol had a statistically significant hazard ratio above 1 for both OS (HR = 1.76; *p* = 4.77 × 10^−3^) and PFI (HR = 1.89; *p* = 1.25 × 10^−4^) (Table 1, Fig. 3a,b). The Prol survival associations were even more pronounced after stratifying by quartile and remained significant after adjusting for tumor stage (Table 1). As expected, the other cell-types did not significantly decrease OS or PFI in TCGA (Additional file 6: Table S5). These results suggest that a high estimated Prol cell-type proportion is associated with poor survival outcomes and plays a key role in HCC tumor aggressiveness.

**Table 1:** Increased abundance of the tumor-associated cell-type Prol is associated with a worse prognosis both in the TCGA and LCI cohorts

Cohort	Event	Prol model	Multivariable HR	95% CI	***p***-value
TCGA	OS	Median	1.76	1.19–2.61	4.77 × 10^−3^
TCGA	OS	Median adj. stage	1.52	1.02–2.26	4.20 × 10^−2^
TCGA	OS	Quartile	3.25	1.84–5.72	4.62 × 10^−5^
TCGA	PFI	Median	1.89	1.37–2.63	1.25 × 10^−4^
TCGA	PFI	Median adj. stage	1.73	1.24–2.41	1.14 × 10^−3^
TCGA	PFI	Quartile	2.85	1.76–4.63	2.14 × 10^−5^
LCI	OS	Median	1.79	1.16–2.76	8.79 × 10^−3^
LCI	OS	Median adj. stage	1.67	1.07–2.60	2.34 × 10^−2^

Hazard ratios of overall survival and progression free interval based on the Prol cell-type proportion in the TCGA HCC cases (*n* = 361) and hazard ratios of overall survival in the LCI HCC cases (*n* = 221) show that an increased abundance of Prol is associated with decreased survival. Cox proportional hazard regression was performed for the event and model indicated. The Prol model indicates the predictor tested. The median model stratifies the cases into low and high abundance groups based on whether the individual’s estimated Prol proportion was below or above the median, respectively. The median adjusted (adj.) stage results are obtained by including in the median model a covariate for the low and high AJCC tumor stage status, where stage I and II form the low stage and stage III and IV form the high stage. The quartile model tests low and high abundance groups by splitting participants below and above the 25th and 75th percentile of Prol proportion estimates, respectively. All tests in TCGA were adjusted for age, sex, and ethnicity. All tests in LCI were adjusted for age and sex. Unadjusted *p*-values are shown. *HR* indicates hazard ratio, *CI* confidence interval, *OS* overall survival, *PFI* progression free interval

Next, we sought to replicate the effect of the HCC risk cell-type Prol on survival in the LCI cohort. As OS was the only available overlapping outcome in LCI, we used OS for our validation analysis. We performed Cox proportional hazards regression adjusting for age and sex. Testing the effect of the Prol cell-type on OS in LCI resulted in a significant hazard ratio (HR = 1.79; *p* = 8.79 × 10^−3^) (Table 1 and Fig. 3c) and remained significant after adjusting for stage (HR = 1.67; *p* = 2.34 × 10^−2^). This result replicated our finding observed in TCGA, demonstrating that a higher Prol is associated with a worse OS outcome in LCI as well. Taken together, the negative link between the tumor Prol cell-type and survival is robust and reproducible across independent HCC cohorts.

We again sought to validate our proportion-based results at the gene level. To do so, we analyzed the relationship between survival outcomes and gene expression of individual cell-type markers. We first performed Cox proportional hazards regression adjusting for age, sex, and ethnicity for all expressed genes in the TCGA HCC liver expression data for OS and PFI as outcomes. We observed that a higher number of Prol-specific marker genes (log fold change > 0.5) had a hazard ratio over 1 for OS (71.7%) and PFI (58.7%) compared to those of all other cell-types (Fig. 3d,e). Additionally, 23.9% and 17.4% of Prol markers had a genome-wide significant hazard ratio for OS and PFI, respectively, all of which were associated with a worse prognosis (Additional file 1: Fig. S8a,b). To replicate these findings, we performed Cox proportional hazards regression for OS in the LCI cohort. Although we did not observe a notable number of genome-wide significant effects, we found a similar enrichment of Prol marker genes with a hazard ratio over 1 for OS (63.6%) (Fig. 3f and Additional file 1: Fig. S8c). These marker gene results further support the conclusion that the Prol cell-type itself is associated with survival.

Finally, we evaluated the cell-type enrichment for all genes with a significant association with OS and PFI in the bulk TCGA cohort and with OS in the LCI cohort. Cells/nuclei in the single-cell level data were assigned survival-decreasing module scores using Seurat for expression of the 740 and 528 genes with a significant hazard ratio above 1 for OS and PFI in the TCGA bulk RNA-seq data, respectively (FDR adjusted *p* < 0.05). We found that the Prol nuclei/cells had the highest average OS-decreasing (Wilcoxon *p* < 2.2 × 10^−16^) and PFI-decreasing (Wilcoxon *p* < 2.2 × 10^−16^) scores (Fig. 3g, h and Fig. 3j, k), indicating that Prol over-expresses DE genes associated with poor survival outcomes in TCGA more prominently than all other cell-types. In order to replicate these results in the LCI cohort, we scored cells/nuclei for expression of the 36 genes that among all genes had a significant hazard ratio above 1 for OS in LCI (FDR adjusted *p* < 0.05). Again, the Prol cluster had the highest average OS-decreasing score from the LCI association results (Wilcoxon *p* = 2.98 × 10^−151^) (Fig. 3i, l). Thus, by taking all genome-wide significant results in an unbiased manner, we highlight the Prol cell-type in poor survival outcomes. Overall, our bulk-based single cell level findings, showing that Prol nuclei/cells significantly over-express both tumor-elevated bulk DE genes (Fig. 2e–h) and survival-decreasing bulk DE genes (Fig. 3g–l), support the association of the Prol cell-type with HCC and worse survival independently from our decomposition analysis.

### Somatic TP53 mutations are associated with increased proportions of the Prol cell-type in HCC

**Fig. 4:**
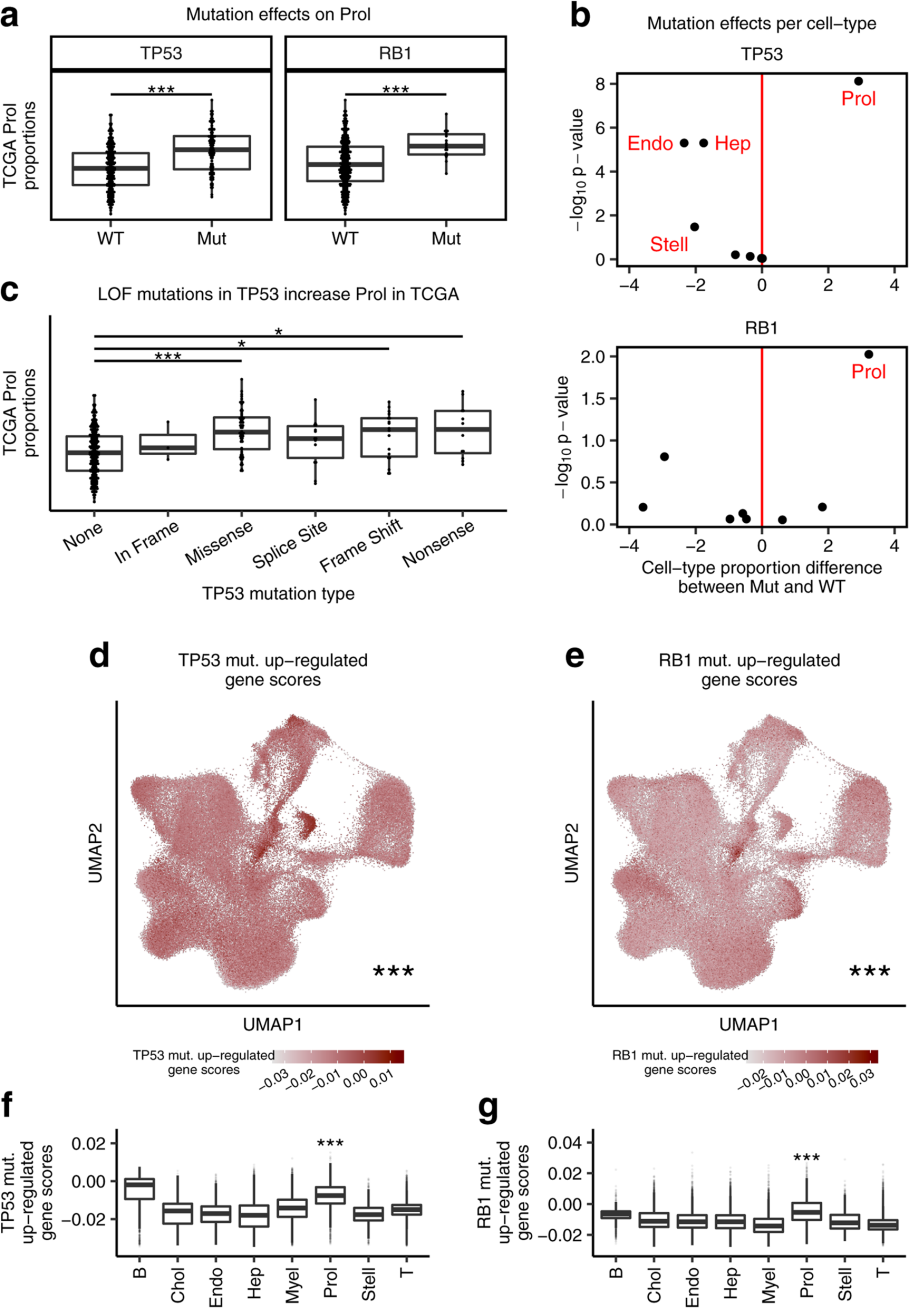
Associations between estimated cell-type proportions and somatic mutations in the TCGA cohort link *TP53* and *RB1* mutations to increased Prol abundance. Mutations associated with changes in the bulk TCGA liver proportion estimates of the Prol cell-type. **a** Prol proportion estimates are significantly higher in the HCC cases harboring a mutation (Mut) in *TP53* (left panel) and *RB1* (right panel) compared to those with both wildtype (WT) alleles. **b** The Prol cell type is highlighted as the only cell-type significantly increased in HCC cases with Mut *TP53* and Mut *RB1*. Differential abundance for the 8 cell-types testing for differences in proportions between Mut vs. WT *TP53* (top panel) and *RB1* (bottom panel) cases. Differential abundance was performed with a Wilcoxon test (*n* = 357 tumor samples). The difference in means of the scaled proportions is plotted in the *x*-axis and the -log10 *p*-value in the *y*-axis. The vertical red line (*x* = 0) indicates no difference. **c** Prol proportion estimates are plotted against no *TP53* mutation (None) and different *TP53* mutation types. Prol estimates are significantly increased in individuals with loss of function (LOF) mutations in *TP53*. **d**–**g** The cells/nuclei in the Prol cell-type significantly express mutation-upregulated genes, as shown by the droplet module scores of mutation upregulated genes for the indicated mutation in TCGA. Mutation upregulated genes were derived by running genome-wide differential expression (DE) between patients with and without a somatic mutation in the indicated gene and taking those over-expressed in HCC patients harboring a mutation and with an FDR-adjusted *p* value less than 0.05. Droplet module scores were calculated by comparing the average expression of mutation upregulated genes to a background set of genes. **d**, **e** UMAP of the single-cell-level data showing droplets colored by scores for genes upregulated in patients with (**d)** *TP53* and (**e)** *RB1* mutations. **f**, **g** Bar plots of the (**e)** *TP53* mutation upregulated scores and (**g)** *RB1* mutation upregulated scores separated by cell-type. **d**, **g** Asterisks denote a significant increase in mutation upregulated gene scores between Prol and non-Prol cells/nuclei as assessed by a Wilcoxon test. Significance levels for nominal *p*-values in (**a**, **c**, **d**-**g)**: *p < 0.05, **p < 0.005, ***p < 0.0005

Somatic mutations in HCC have been characterized in several cohorts, and although heterogeneous, these studies have identified commonly mutated driver genes [5]. However, it has remained elusive whether somatic mutations can lead to specific tumor cell-type expansions or depletions. Therefore, we performed associations between cell-type profiles against mutations in the 69 significantly mutated genes that have previously been characterized in TCGA HCCs (https://gdac.broadinstitute.org). We identified 3 genes associated with a higher cell-type abundance (Wilcoxon adjusted *p* < 0.05) (Additional file 7: Table S6). Among these, mutations in *TP53* (Wilcoxon adjusted *p* = 7.58 × 10^−9^) and in *RB1* (Wilcoxon adjusted *p* = 9.45 × 10^−3^) led to a significant increase in the estimated proportions of the Prol tumor cell-type (Fig. 4a and Additional file 1: Fig. S9). Furthermore, Prol was the only significantly increased cell-type in individuals with *TP53* mutations or *RB1* mutations (Fig. 4b). Interestingly, we also observed that *BAP1* mutations are associated with an increase in cholangiocyte proportion estimates. *BAP1* has been shown to be frequently inactivated in cholangiocarcinomas [62].

Many mutations in *TP53* are known to lead to a loss of the tumor suppressor function of p53 and consequently uncontrolled cell growth [63]. We therefore tested the effect of distinct *TP53* mutation types on Prol abundance. We observed that *TP53* missense, frame shift, and nonsense mutations led to significantly higher proportions of Prol (Fig. 4c). While frame shift and nonsense mutations are likely to lead to a total loss of function, missense mutations in *TP53* have been found to occur mainly in the DNA-binding domain of the protein, also leading to a loss of its tumor suppressor function [63]. To further investigate whether the Prol cell-type is the main consequence of *TP53* mutations, we first identified 1358 mut. over-expressed genes with significant log fold changes greater than 0.5 between mutation (mut.) carriers and wildtype (WT) cases in TCGA. We then assigned module scores to droplets in the single-cell level data based on expression of these mut. upregulated genes. We found that the Prol cells/nuclei had significantly higher *TP53* mutation scores than all other cells/nuclei (Wilcoxon *p* < 2.2 × 10^−16^) (Fig. 4d, f). We performed the same analysis for 774 *RB1* DE genes and found a similar enrichment of mut. upregulated gene scores in Prol droplets (Wilcoxon *p* < 2.2 × 10^−16^) (Fig. 4e, g). Overall, these results suggest that distinct somatic mutations can lead to a tumor cell-type expansion and highlight the role of *TP53* mutations in proliferation and uncontrolled cell growth.

## Discussion

We developed a new framework using comprehensive single cell level reference data from multiple etiologies of HCC, adjacent non-tumor, and normal liver tissue to decompose cell-types in liver bulk RNA-seq and microarray expression data generated from HCC and adjacent non-tumor tissue in the TCGA and LCI cohorts. This integrative transcriptomics framework identified an HCC-associated proliferative cell-type, Prol, the high proportion of which in HCC tumors is associated with significantly worse survival outcomes. Noteworthy, we first observed this survival effect in TCGA, and then replicated our finding in LCI. Our results should be robust not only because we replicated our findings in an independent cohort, but also because they do not depend on the technology used to measure single cell and tissue-level gene expression in the liver, given that both scRNA-seq and snRNA-seq were used to build the reference data set and both bulk RNA-seq in TCGA and microarray technology in LCI were used to decompose the cell-types in the liver tissue. Furthermore, our reciprocal module score analyses show that Prol nuclei/cells significantly over-express both tumor-elevated DE genes and survival-decreasing DE genes obtained from the bulk expression data in the TCGA and LCI cohorts. Thus, these bulk-based single cell level results further support the association of the Prol cell-type with HCC and worse survival independently from the decomposition analysis. When searching for mutated driver genes of the HCC cell-types, we found that among 69 genes with somatic mutations catalogued in TCGA earlier (https://gdac.broadinstitute.org), Prol is the only significantly increased cell-type in individuals with *TP53* and *RB1* mutations. Thus, we show that mutations in these tumor suppressor genes are associated with the expansion of the tumor-associated Prol cell-type in HCC.

Exploring cell-type heterogeneity provides a novel avenue to study microenvironment in cancer cells. The notion that tumor microenvironment, specifically of immune cells, may affect tumor progression and affect survival first stemmed from non-HCC studies, such as ovarian tumors [64]. This was subsequently investigated in HCC [19, 65], which has clinical implications given that only ~ 18% of patients responded to therapies targeted to immune-dependent pathways with checkpoint inhibitors (programmed death 1 or PD1) in early clinical trials [66]. Losic and colleagues used scRNA-seq from 2 patients across multiple regions within the same tumor to demonstrate tumor heterogeneity, which provided early evidence that the immune microenvironment is heterogeneous between patients and within samples [19]. In line with these studies, we observed similar heterogeneity in cell-type composition within HCC patients from multiple etiologies. Using single cell level data generated by both scRNA-seq and snRNA-seq, we found patient-specific clusters when not correcting for this heterogeneity with integration (Additional file 1: Fig. S3).

Large existing cohorts, such as TCGA [26] and LCI [27], provide invaluable tools to the research community. Accordingly, we leveraged our integrated liver single cell level data to identify cell-types associated with HCC and their clinically significant outcomes in TCGA and LCI, both with long-term follow-up data. The systematic identification of the Prol cell-type across the single cell level reference data with multiple etiologies of HCC, the TCGA cohort (mostly viral etiologies with HBV and HCV), and the LCI cohort (HBV-predominant origin of HCC) suggests universal points of convergence in HCC pathogenesis that can be further investigated at the single cell level. Understanding tumor biology at the cell-type level instead of the bulk tissue level provides more insight into the underlying tumor biology [67]. Several of the Prol marker genes have previously been associated with poor survival outcomes [52–59, 68]; however, our study discovered that these genes form a distinct HCC-associated cell-type. Furthermore, we discovered that somatic mutations in *TP53* and *RB1* are associated with increased Prol proportions in HCC. Interestingly, differences in somatic mutations have also been observed in various etiologies of HCC, with the *TP53* mutations being linked to viral and alcohol etiologies of HCC [69] (similar to the patient composition of the TCGA and LCI cohorts), while *ACVR2A* (activin A receptor type 2A) mutations have been more commonly found in NASH-HCC [69].

Previous studies have identified molecular sub-classes of HCC that correlate with tumor phenotypes and clinical outcomes [6, 26, 70–72]. About half of all HCCs consist of the proliferative sub-class that predominantly have *TP53* mutations [6], which we also identified as significant mutations in our cell-type analyses. Our data suggest that somatic mutations in the tumor suppressor gene, *TP53*, result in dysregulation of mitosis and cell-cycle pathways, in line with their enrichment in the Prol cell-type. Consistent with our findings, in an independent study, the histone protein, H2AFZ that we identified as a marker gene in Prol, was associated with cell cycle genes and reported to be regulated by TP53 in HCC [59]. Overall, our integrative approach identified a cell-type with somatic mutations in a tumor suppressor gene that is significantly associated with worse overall survival. These results may improve current HCC subclassification and provide insight into co-dependent biological mechanisms of HCC.

Several of the genes identified in the Prol cell-type have previously been associated with poor overall or recurrence-free survival outcomes in HCC, including *PTMA* [52, 68], *HMGB2* [53], *HMGB1* [54], *H2AFZ* [59], *GAPDH* [55], *TUBB* [57], *STMN1* [56], and *TUBA1B* [58]. However, despite this growing body of literature identifying individual HCC genes with prognostic potential in the TCGA and other cohorts, our study used a single cell level-based decomposition approach to identify an HCC-associated cell-type, the proportion of which is significantly increased in HCC tumors with poor survival. The Prol cell-type suggests uncontrolled mitosis and cell-cycle dysregulation as converging mechanisms for worse survival. Furthermore, the Prol cell-type not only contains previously known HCC genes [52–59, 68], but also provides new targets, including *HMGN2*, *RARRES2*, and *HIST1H4C* that have not been explored yet. Overall, our integrative multi-cohort approach provided hundreds of Prol cell-type marker genes, which can be used to advance our understanding of the complex HCC biology in future studies.

Given the poor survival outcomes in patients diagnosed with HCC [3, 66], it is critical to further our understanding of factors affecting survival. We demonstrate that the use of cell-type markers could be of clinical utility as a potential future biomarker to guide treatment options and determining clinical outcomes. Current clinical prognostic tools of HCC mostly rely on the number and size of tumors, AFP, the presence of underlying chronic liver disease, and the patient’s medical status. The use of cell-type markers as a tool to understand tumor biology can improve current clinical practice. Our Prol marker genes could serve as a basis for developing new expression-based prognostic technologies. For example, quantitative PCR could be used to rapidly perform predictive gene expression panel tests [73]. As RNA sequencing matures, clinical labs can detect global gene expression patterns with prognostic value [74]. Assays such as these could measure Prol markers to evaluate the abundance of the two cell types and test if they predict clinical outcomes. Whether this cell-type is prognostic for HCC recurrence post resections or liver transplantation would also need to be determined. Our pipeline utilizing single cell level reference data to decompose cell-types in bulk RNA-seq can also be applied to other malignancies that have an admixture of heterogeneous cells to identify predominant cancer cell-types.

Although this study improves our understanding of new HCC cell-types with a potential for clinical implications, it is not without limitations. As HCC prevalence continues to rise and liver transplantation allocation policies are changing [75], larger studies with different HCC etiologies are needed in cirrhosis and non-cirrhosis backgrounds, especially given the observed differences in treatment responses [22]. In addition, cell-type changes in recurrent HCCs would have to be investigated in future studies. It should also be noted that although our survival analyses in TCGA discovered the significance of the Prol cell-type in OS and PFI, even after adjusting for tumor stage, other clinically relevant factors in HCC outcomes, including AFP levels, extent of chronic liver disease, presence of lymph vascular invasion on histopathology, and tumor size could not be explored in our models because up to 35% of the 361 individuals had missing data for these parameters. Thus, future studies are warranted to assess their correlations with the Prol tumor-associated cell-type.

## Conclusions

In conclusion, using comprehensive single cell level reference data to decompose cell-types in the TCGA and LCI liver bulk tissue cohorts, we discover the important role of the previously unknown Prol cell-type in HCC and survival outcomes in TCGA, which replicated in LCI. We also linked somatic mutations in the tumor suppressors *TP53* and *RB1* to Prol cell-type expansion in HCC. Our integrative transcriptomics pipeline can be extrapolated to other cancer cohorts to identify key tumor cell-types using single cell level samples as the cell-type reference data. The detection of tissue-specific and cancer-associated cell-types can advance our understanding of tumor biology with a great potential for biomarker discovery in larger, prospective validation studies.

## Supplementary Information

**Additional file 1: Supplementary figures (Fig. S1-S9).**

**Additional file 2: Table S1.** Subcell-type marker genes.

**Additional file 3: Table S2.** Reactome gene set enrichment analysis of the subcell-type marker genes.

**Additional file 4: Table S3.** Bisque marker genes used for decomposition.

**Additional file 5: Table S4.** Differential proportion analysis between HCC tumor and adjacent non-tumor tissue in the TCGA and LCI liver bulk tissue cohorts.

**Additional file 6: Table S5.** Associations of main cell-type proportions with survival outcomes in TCGA.

**Additional file 7: Table S6.** Associations between main cell-type proportions and somatic mutations in TCGA.

## Data Availability

Raw snRNA-seq counts for the NAFLD-related HCC cohort [25] are available from NIH GEO under accession number GSE189175. Liver HCC scRNA-seq data from Sharma et al. [8] are available from https://data.mendeley.com/datasets/6wmzcskt6k/1. Read counts for the scRNA-seq of the 9 normal liver samples from Aizarani et al. [7] are available from NIH GEO under the accession number GSE124395. The TCGA [26] data are available for downloading at (https://portal.gdc.cancer.gov/projects/TCGA-LIHC) and (http://gdac.broadinstitute.org). The LCI [27] data are available for downloading from NIH GEO under accession number GSE14520. The code used for the analyses in this manuscript can be found in https://github.com/marcalva/hcc_sc_2022 [76].
