# Supplementary material for: Human liver single nucleus and single cell RNA sequencing identify a hepatocellular carcinoma-associated cell-type affecting survival
Source: Genome Med. 2022 May 17;14:50. doi: 10.1186/s13073-022-01055-5 (PMC9115949; doi:10.1186/s13073-022-01055-5)
Supplement: Supplementary file 1 — Additional file 1: Supplementary figures (Fig. S1-S9). [file 13073_2022_1055_MOESM1_ESM.pdf]

Supplementary Information

Human liver single nucleus and single cell RNA-sequencing identify a hepatocellular carcinoma-associated cell-type affecting survival

Marcus Alvarez[1]# malvarez@mednet.ucla.edu

Jihane N Benhammou[2,3]# jbenhammou@mednet.ucla.edu

Nicholas Darci-Maher[1] ndarci@mednet.ucla.edu

Samuel W French[4] sfrench@mednet.ucla.edu

Steven B Han[5] sbhan@mednet.ucla.edu

Janet S Sinsheimer[1,6,7] jsinshei@ucla.edu

Vatche G Agopian[8] vagopian@mednet.ucla.edu

Joseph R Pisegna[1,3] jpisegna@mednet.ucla.edu

Päivi Pajukanta[1,7,9*] ppajukanta@mednet.ucla.edu

[1]Department of Human Genetics, David Geffen School of Medicine at UCLA, Los Angeles, CA, USA

[2]Vatche and Tamar Manoukian Division of Digestive Diseases, David Geffen School of Medicine at UCLA, Los Angeles, CA, USA

[3]Division of Gastroenterology, Hepatology and Parenteral Nutrition, Department of Medicine, VA Greater Los Angeles Healthcare System

[4]Department of Pathology, David Geffen School of Medicine at UCLA, Los Angeles, CA, USA

[5]Department of Medicine, David Geffen School of Medicine, University of California, Los Angeles, CA, USA
[6]Department of Computational Medicine, UCLA, Los Angeles, CA 90095, USA
[7]Bioinformatics Interdepartmental Program, UCLA, Los Angeles, CA, USA
[8]Dumont-UCLA Transplant and Liver Cancer Centers, Department of Surgery, David Geffen School of Medicine at UCLA, Los Angeles, CA
[9]Institute for Precision Health, David Geffen School of Medicine at UCLA, Los Angeles, CA, USA
#Equal contribution

*Correspondence:
Päivi Pajukanta, MD, PhD
Professor of Human Genetics
Diller-von Furstenberg Family Endowed Chair in Precision Clinical Genomics
Vice Chair, Department of Human Genetics
Director of Cardiometabolic Genomics, Institute for Precision Health
Director of Genetics and Genomics PhD Program
David Geffen School of Medicine at UCLA
University of California, Los Angeles (UCLA)
Gonda Center, Room 6357B
695 Charles E. Young Drive South
Los Angeles, California 90095-7088, USA
Email: ppajukanta@mednet.ucla.edu

Supplementary Figures

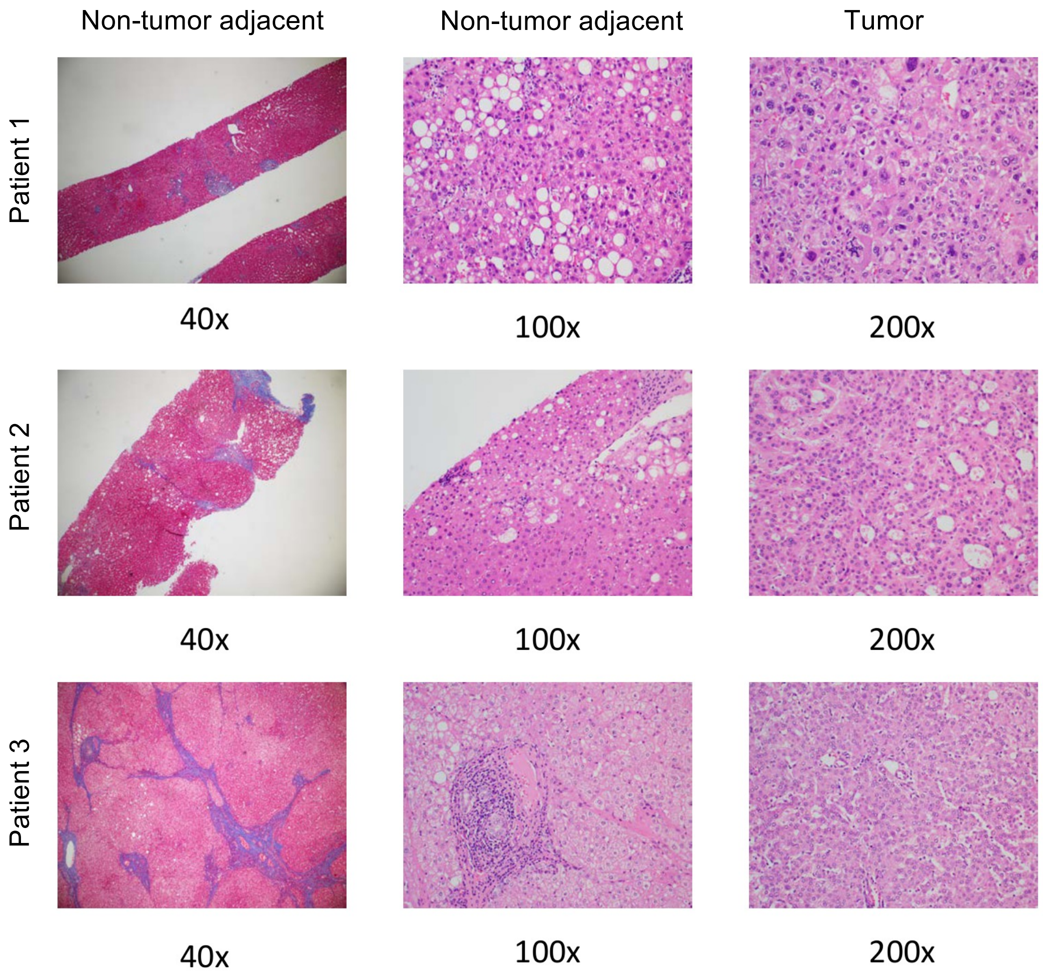

Fig. S1. Histopathology of tumor and adjacent non-tumor biopsies in the 3 NAFLD-related HCC cases.

Histopathology slides using hematoxylin and eosin and trichrome stains demonstrate tumor and patient heterogeneity.

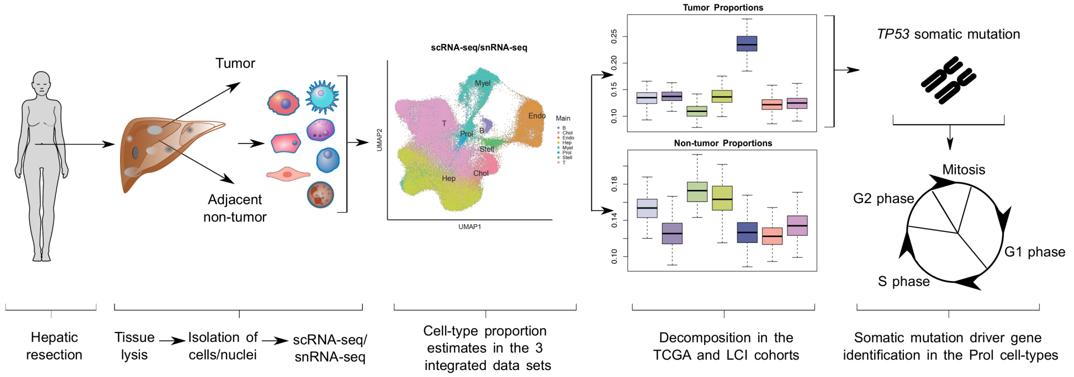

Fig. S2. Overview of study design to profile cell composition changes in HCC.

Single-cell and single-nucleus RNA-seq (scRNA-seq and snRNA-seq) were used to profile cell-type transcriptomes in human livers from non-HCC, HCC tumor, and adjacent non-tumor tissue. We performed snRNA-seq on tumor and adjacent non-tumor biopsies from three patients with fatty liver related HCC. Our snRNA-seq was integrated with two single-cell RNA-seq data sets from Aizarani *et al.* [7] and Sharma *et al.* [8] to characterize transcriptional profiles across various etiologies of HCC. The identified cell-types and their gene expression were used to estimate their proportions in larger bulk liver HCC RNA-seq cohorts with survival outcome data. These analyses highlighted the role of a tumor-associated mitotic cell-type Prol, associated with survival outcomes and *TP53* mutations.

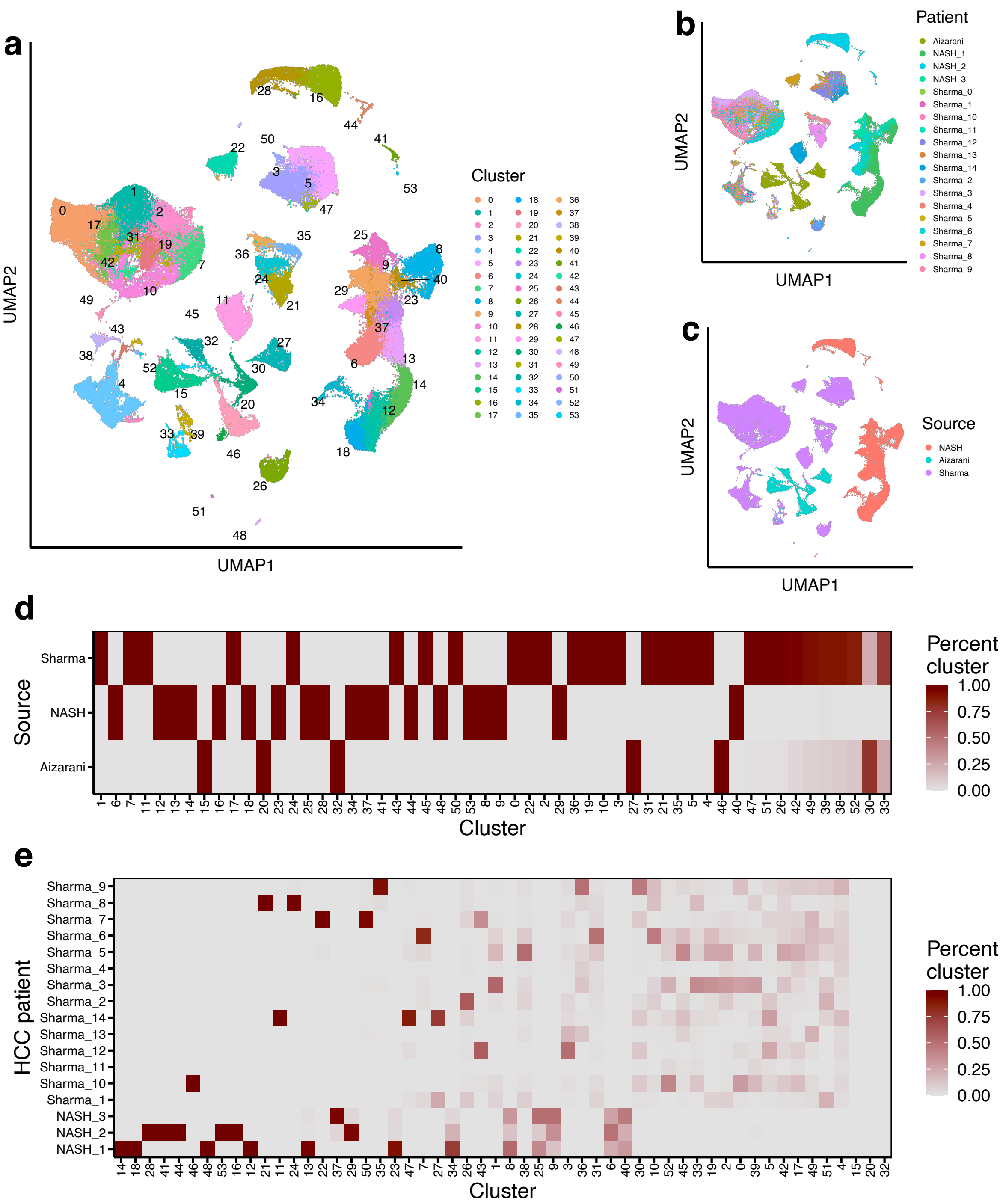

Fig. S3. Un-integrated merging of the three single cell level cohorts results in cohort- and patient-specific batch effects.

a-c, UMAP plots of the three single cell level cohorts after merging without integration. Raw counts were normalized with sctransform [37], and clustering was performed on the PCs with a resolution of 1.0. Cells and nuclei are colored by **a,** cluster, **b,** patient, and **c,** cohort (source). **d,e,** The heatmap plots show the prevalence of cohort and patient effects in the merged data without integration. Each heatmap indicates the proportion of droplets in a cluster that originate from **d,** cohort (source) and **e,** HCC patient (excluding the Aizarani *et al.* cohort [7] that comprises only healthy controls and the healthy control from the Sharma *et al*. data [8]). For each of the 54 clusters, the column proportions sum to 1. Cells and nuclei from a cohort cluster together, indicating the presence batch effects, while several clusters show patient-specific effects and suggest inter-patient heterogeneity.

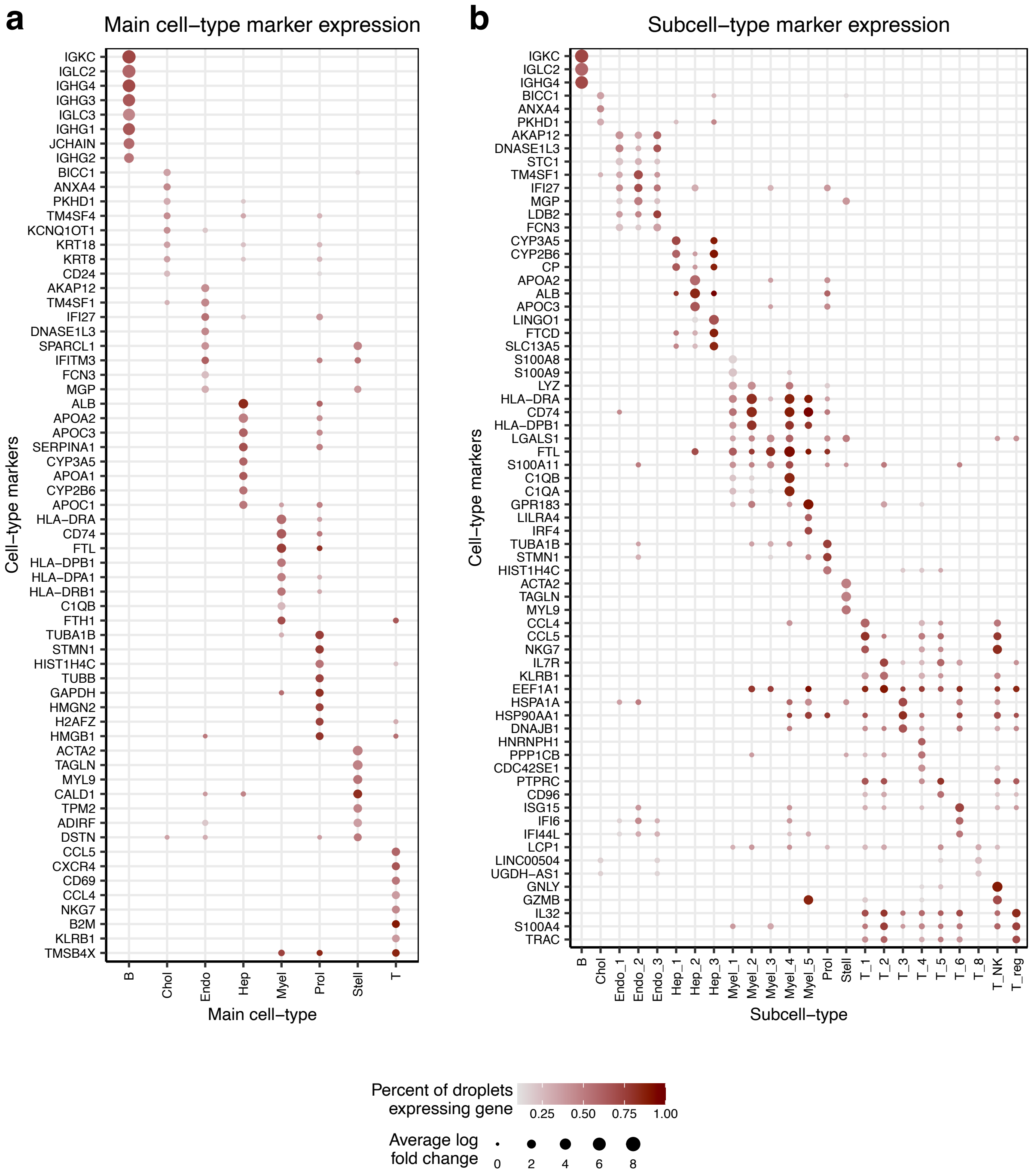

Fig. S4. Expression of top up-regulated marker genes across cell-types in the integrated single cell level data supports the functional identity of the assigned cell-types.

a,b, Expression of the top marker genes for **a,** main cell-types and **b,** subcell-types supports the functional identity of the assigned cell-types. The **a,** top 8 marker genes per main cell-type and **b,** top 3 marker genes per subcell-type are shown. A logistic regression in Seurat [38] was used to test the difference in expression between droplets in the indicated main cell-type/subcell-type and all other droplets. The percent of droplets expressing the marker gene indicates the percent which have at least one UMI aligned to the gene. The average log fold change indicates the $\log_2$ fold change of the average expression of the main cell-type/subcell-type droplets over the average expression of all other droplets. Main cell-types were assigned by merging subcell-types based on their major lineage. Cells and nuclei from T_7 contain no statistically significant marker genes.

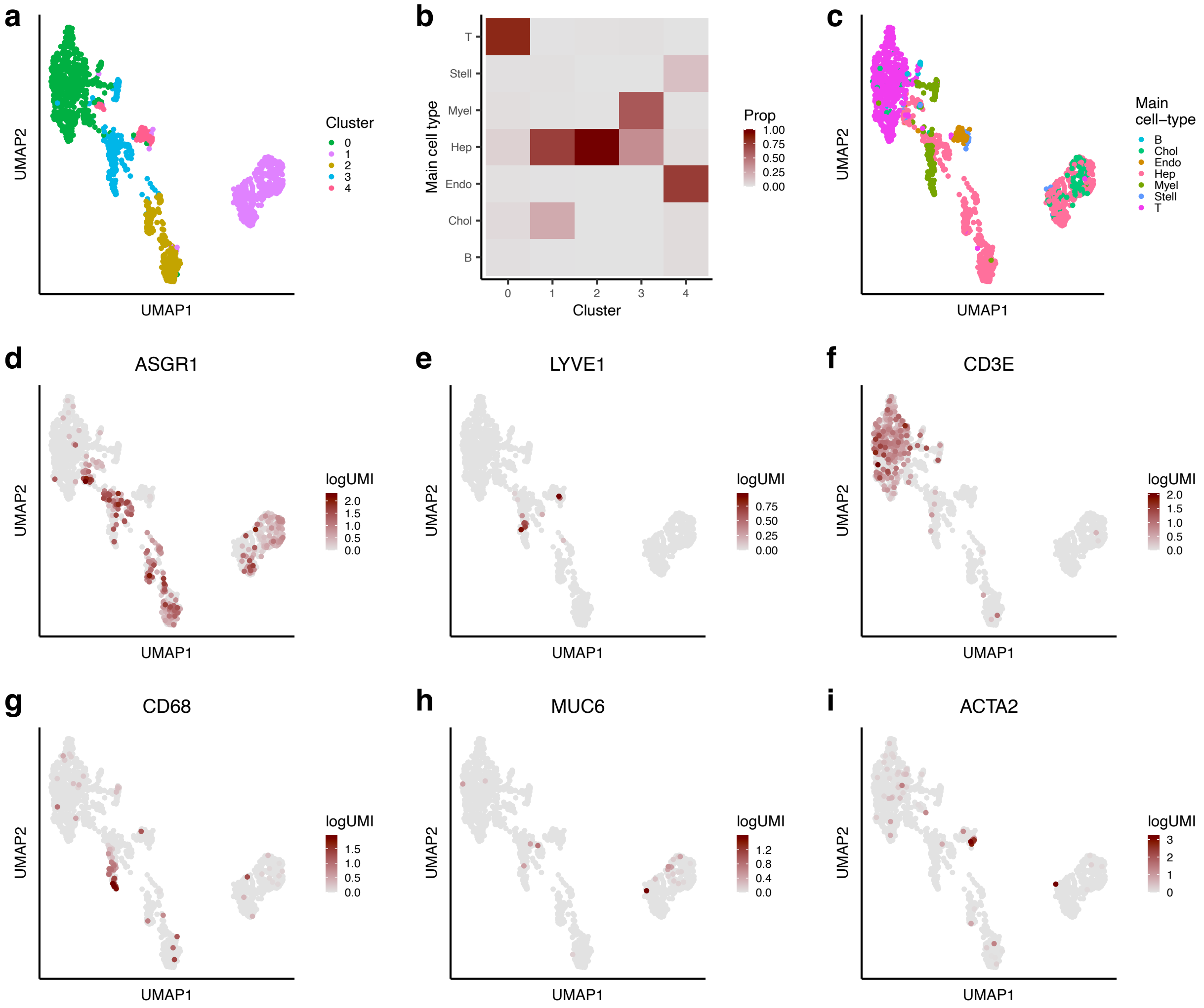

Fig. S5. Cells and nuclei from the Prol cell-type subcluster into main liver cell-types.

a, UMAP of cells and nuclei from Prol colored by subcluster. The 1,743 droplets from the Prol cluster identified in the full single-cell-level data set were subclustered after sctransform [37] and CCA integration by cohort using a resolution of 0.2 [38]. **b,** Proportion of cells/nuclei in the Prol cell-type classified into all other major cell-types. Classifications were performed using SingleR [43] with a reference trained on the full data set that excluded the Prol cluster. **c,** UMAP of Prol cells and nuclei colored by SingleR classification to all other main cell-types (consisting of 41.7%

hepatocyte, 33.8% T, 9.9% myeloid, 7.7% cholangiocyte, 4.4% endothelial, 1.6% stellate, and 0.9% B cells). **d-i,** UMAP of Prol cells/nuclei colored by log-normalized gene expression. Expression of the marker genes **d,** *ASGR1* (Hepatocyte), **e,** *LYVE1* (Endothelial), **f,** *CD3E* (T), **g,** *CD68* (Macrophage), **h,** *MUC6* (Cholangiocyte), **i,** *ACTA2* (Stellate) are shown in subclusters.

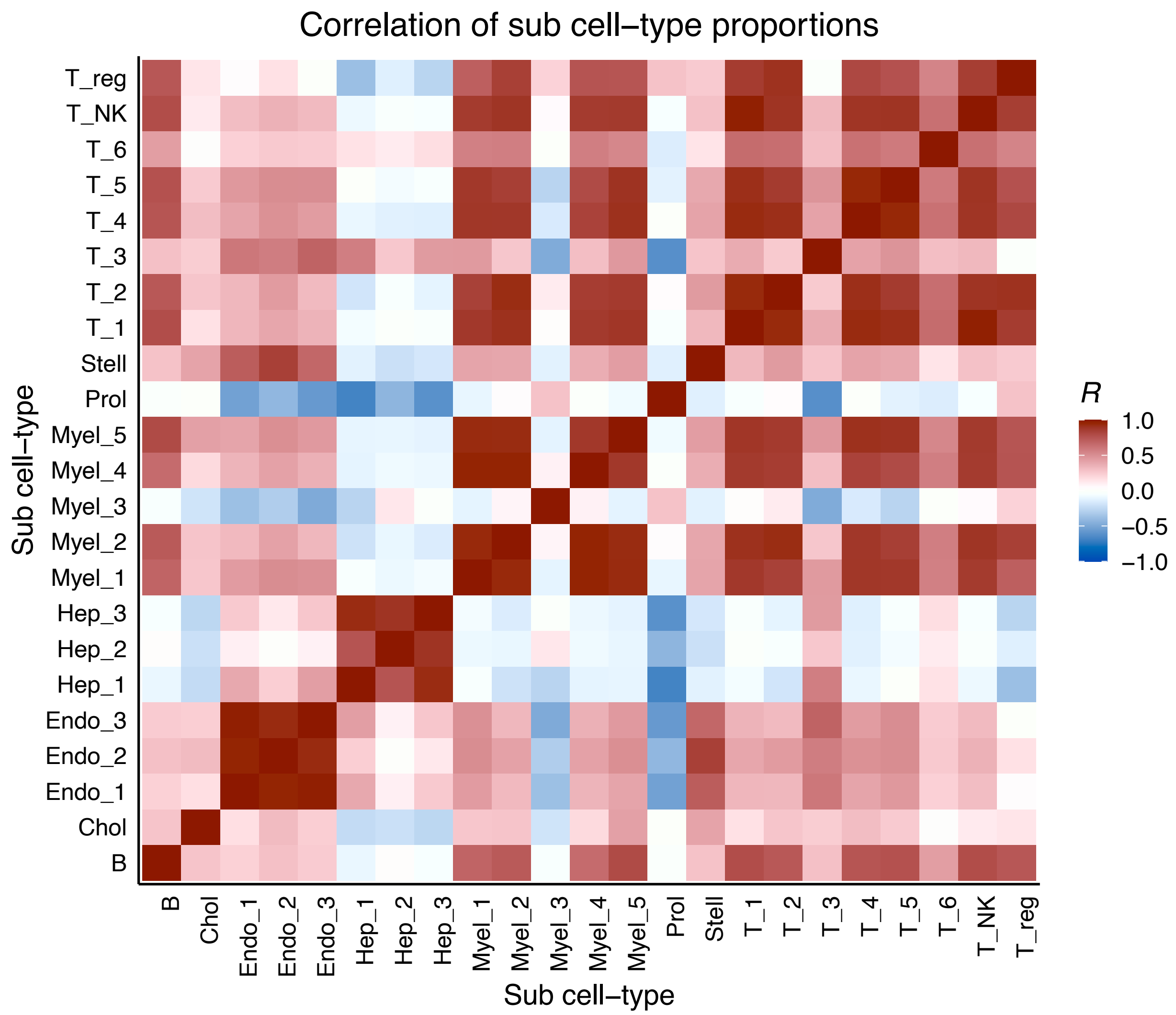

Fig. S6. Proportion estimates for sub cell-types within a main group show high correlation in TCGA.

The heatmap shows the pairwise Pearson correlation coefficients (R) between sub cell-type proportion estimates in TCGA. Proportions were estimated in the 410 bulk liver RNA-seq liver samples using Bisque [14]. Cell-types from the same main group (for example, hepatocytes) show high correlations.

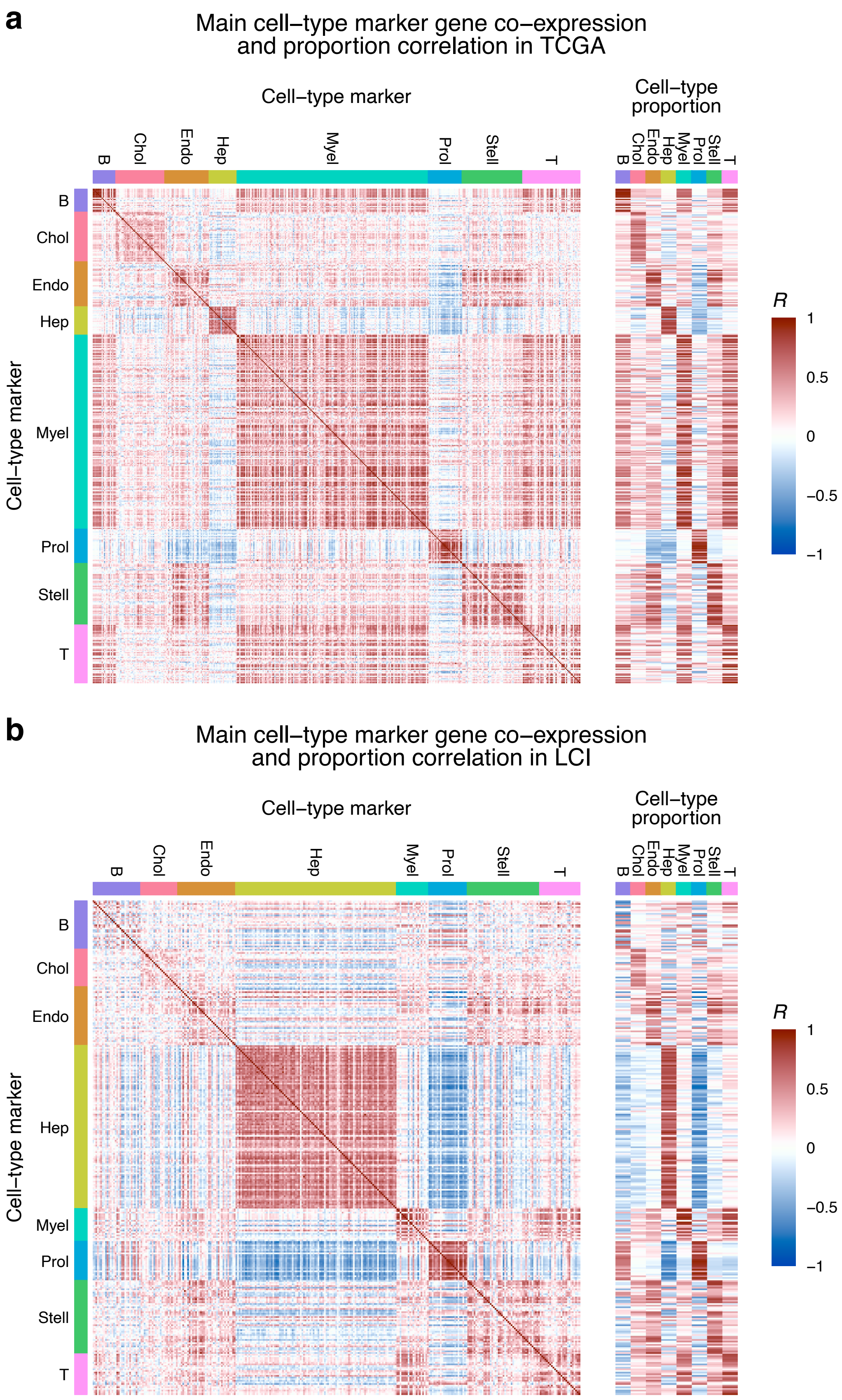
a
Main cell–type marker gene co–expression
and proportion correlation in TCGA
Cell–type marker
Cell–type
proportion
B
Chol
Endo
Hep
Myel
Prol
Stell
T
Cell–type marker
R
1
0.5
0
−0.5
−1
b
Main cell–type marker gene co–expression
and proportion correlation in LCI
Cell–type marker
Cell–type
proportion
B
Chol
Endo
Hep
Myel
Prol
Stell
T
Cell–type marker
R
1
0.5
0
−0.5
−1

Fig. S7. High intra-cell-type co-expression of main cell-type markers supports decomposed proportion estimates in TCGA and LCI.

Marker gene co-expression and proportion correlation for the main cell-types validates the reference-free approach to decompose cell-type frequency estimates. The plots show the co-expression of the top subset of marker genes ordered by cell-type as well as the expression-proportion correlations in **a,** TCGA (n=410) and **b,** LCI (n=430). Each tile displays the Pearson correlation coefficient (R). The left panel shows the correlation between of the expression of marker pairs, where marker genes within the same cell-type display higher co-expression than outside the cell-type. The right panel shows the correlation between the expression of marker genes and proportion estimates. The co-expressed marker genes show high correlations with their cell-type proportion estimates, validating that the proportion estimates are reflective of marker gene RNA abundance. The top subset of single cell markers and proportion estimates were calculated by Bisque [14] in the reference-free decomposition procedure. Marker genes for the B cell-type in the LCI cohort show lower intra-correlations when compared to marker gene sets of the other main cell-types, indicating that their expression is not indicative of B cell abundance.

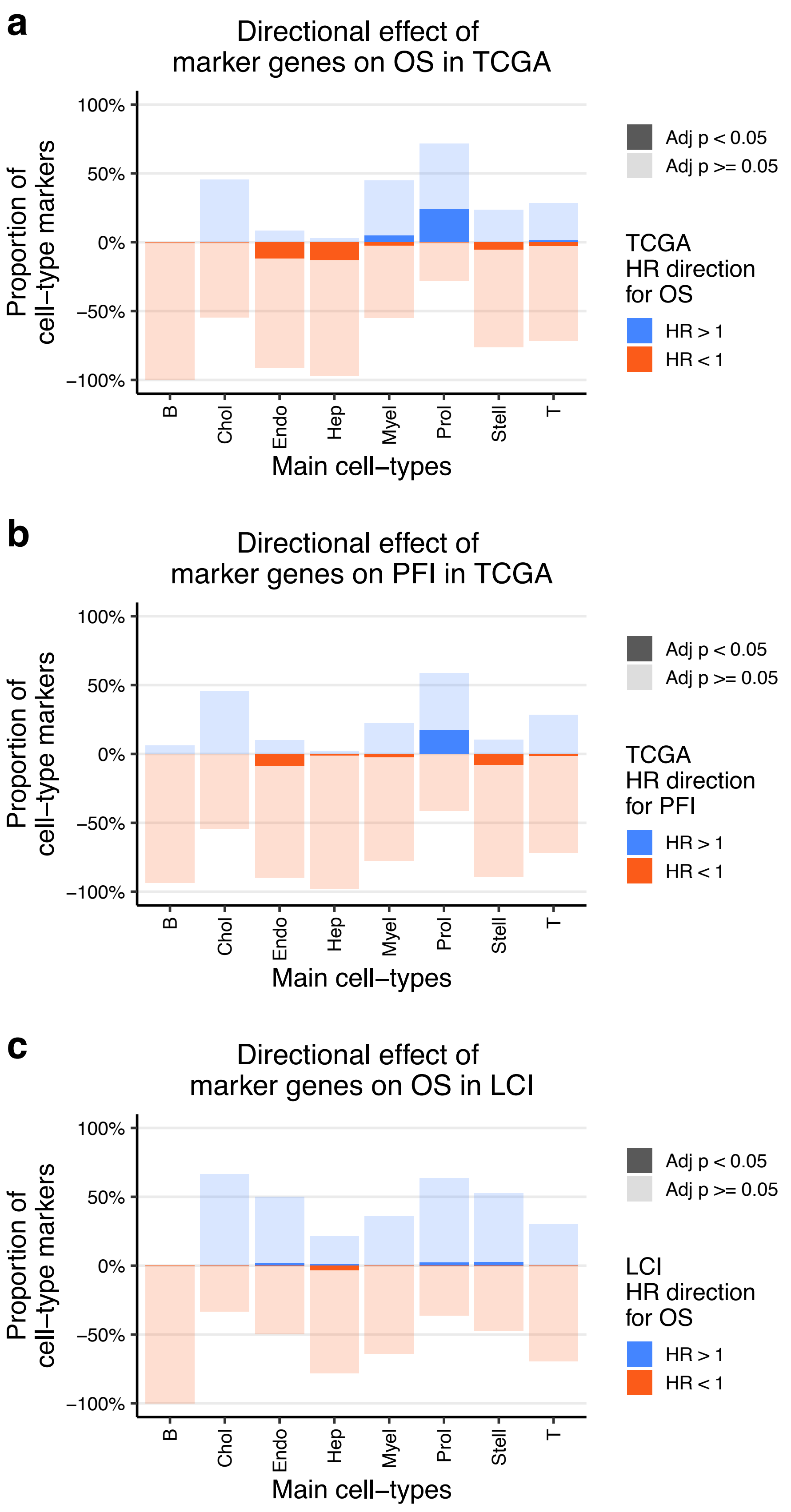
a
Directional effect of
marker genes on OS in TCGA
Proportion of
cell−type markers
100%
50%
0%
−50%
−100%
B
Chol
Endo
Hep
Myel
Prol
Stell
T
Main cell−types
Adj p < 0.05
Adj p >= 0.05
TCGA
HR direction
for OS
HR > 1
HR < 1
b
Directional effect of
marker genes on PFI in TCGA
Proportion of
cell−type markers
100%
50%
0%
−50%
−100%
B
Chol
Endo
Hep
Myel
Prol
Stell
T
Main cell−types
Adj p < 0.05
Adj p >= 0.05
TCGA
HR direction
for PFI
HR > 1
HR < 1
c
Directional effect of
marker genes on OS in LCI
Proportion of
cell−type markers
100%
50%
0%
−50%
−100%
B
Chol
Endo
Hep
Myel
Prol
Stell
T
Main cell−types
Adj p < 0.05
Adj p >= 0.05
LCI
HR direction
for OS
HR > 1
HR < 1

Fig. S8. Expression of Prol marker genes are associated with poor survival outcomes in TCGA and LCI.

a-c, The bar plots show the percent of marker genes that are positively and significantly associated with **a,** overall survival (OS) and **b,** progression free interval (PFI) in TCGA and **c,** OS in LCI. We considered marker genes as those with a log2 fold change (logFC) greater than 0.5 and an FDR-adjusted p-value less than 0.05. For each main cell-type, the percent of its marker genes that decrease survival outcomes (HR > 1) and increase survival outcomes (HR < 1) are shown by color. The percent of these genes that pass genome-wide multiple testing with an FDR-adjusted p-value less than 0.05 are shown by the darker fill for each direction.

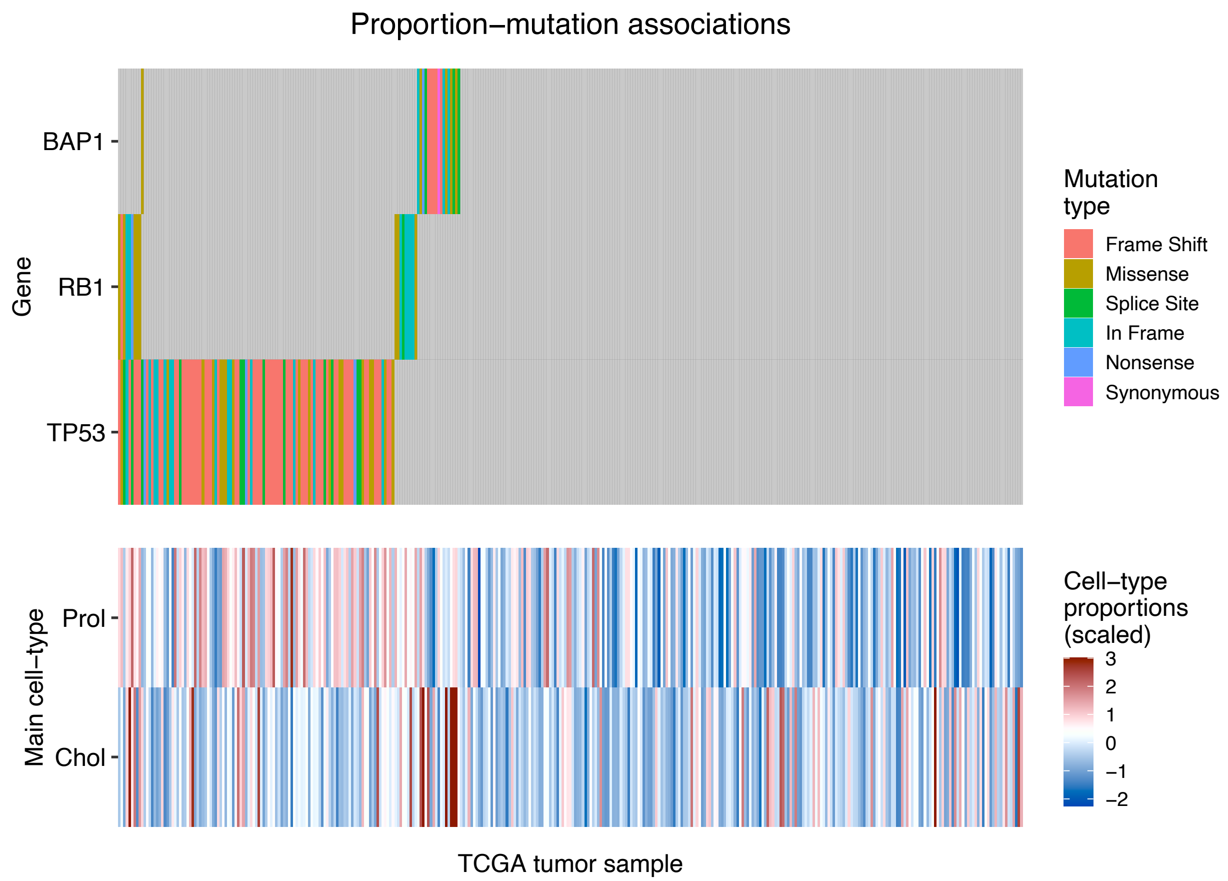

Fig. S9. Prol proportions are increased with *TP53* and *RB1* mutations.

The plot shows the proportion estimates of significantly increased cell-types (bottom) by somatic mutation (top). Proportions were tested for differences between individuals with and without a somatic mutation in significantly mutated HCC genes with a Wilcoxon test (n=357). Significant gene-cell-type pairs with an increase in proportions are shown (FDR-adjusted $p < 0.05$). The top panel shows the somatic mutation (colored by type) present in each of the 357 primary tumor samples, while the bottom panel shows their estimated cell-type proportions (scaled).
